# Metagenomic and geochemical characterization of pockmarked sediments overlaying the Troll petroleum reservoir in the North Sea

**DOI:** 10.1186/1471-2180-12-203

**Published:** 2012-09-11

**Authors:** Othilde Elise Håvelsrud, Thomas HA Haverkamp, Tom Kristensen, Kjetill S Jakobsen, Anne Gunn Rike

**Affiliations:** 1 Norwegian Geotechnical Institute, Sognsveien 72, P.O. Box 3930, Ullevål Stadion N-0806, Oslo, Norway; 2 Department of Molecular Biosciences, University of Oslo, Blindernveien 31, P.O. Box 1041, Blindern N-0316, Oslo, Norway; 3 Microbial Evolution Research Group, MERG, Department of Biology, University of Oslo, Blindernveien 31, P.O. Box 1066, Blindern N-0316, Oslo, Norway; 4 Centre for Evolutionary and Ecological Synthesis (CEES), Department of Biology, University of Oslo, Blindernveien 31, P.O. Box 1066, Blindern N-0316, Oslo, Norway

## Abstract

**Background:**

Pockmarks (depressions in the seabed) have been discovered throughout the world’s oceans and are often related to hydrocarbon seepage. Although high concentrations of pockmarks are present in the seabed overlaying the Troll oil and gas reservoir in the northern North Sea, geological surveys have not detected hydrocarbon seepage in this area at the present time. In this study we have used metagenomics to characterize the prokaryotic communities inhabiting the surface sediments in the Troll area in relation to geochemical parameters, particularly related to hydrocarbon presence. We also investigated the possibility of increased potential for methane oxidation related to the pockmarks. Five metagenomes from pockmarks and plain seabed sediments were sequenced by pyrosequencing (Roche/454) technology. In addition, two metagenomes from seabed sediments geologically unlikely to be influenced by hydrocarbon seepage (the Oslofjord) were included. The taxonomic distribution and metabolic potential of the metagenomes were analyzed by multivariate analysis and statistical comparisons to reveal variation within and between the two sampling areas.

**Results:**

The main difference identified between the two sampling areas was an overabundance of predominantly autotrophic nitrifiers, especially *Nitrosopumilus*, and oligotrophic marine Gammaproteobacteria in the Troll metagenomes compared to the Oslofjord. Increased potential for degradation of hydrocarbons, especially aromatic hydrocarbons, was detected in two of the Troll samples: one pockmark sample and one from the plain seabed. Although presence of methanotrophic organisms was indicated in all samples, no overabundance in pockmark samples compared to the Oslofjord samples supports no, or only low level, methane seepage in the Troll pockmarks at the present time.

**Conclusions:**

Given the relatively low content of total organic carbon and great depths of hydrocarbon containing sediments in the Troll area, it is possible that at least part of the carbon source available for the predominantly autotrophic nitrifiers thriving in this area originates from sequential prokaryotic degradation and oxidation of hydrocarbons to CO_2_. By turning CO_2_ back into organic carbon this subcommunity could play an important environmental role in these dark oligotrophic sediments. The oxidation of ammonia to nitrite and nitrate in this process could further increase the supply of terminal electron acceptors for hydrocarbon degradation.

## Background

Pockmarks, described as craterlike depressions on the seafloor, were first discovered at the Scotian Shelf and are likely to be formed by ascending gas or water [1]. The features have later been discovered throughout the world’s oceans, e.g. the Norwegian continental slope [2], the equatorial West African margin [3], the Bering Sea [4] and the Belfast Bay, Maine [5].

Pockmarks may in some instances be related to active seepage, such as at Gullfaks and Tommeliten (North Sea), where methane is emitted at the seafloor. At these sites anaerobic methanotrophic archaea (ANME) have been found to be important members of the microbial community in the sediments [6,7]. ANME and their sulphate reducing bacterial partners are key players in anaerobic methane oxidation and ubiquitous in all methane environments (e.g. Haakon Mosby Mud Volcano [8], Coal Oil Point seep sediments [9], Eel River sediments [10], Black Sea microbial mats and Hydrate Ridge [11]) [12].

One area characterized by a high density of pockmarks is the seabed overlaying the Troll petroleum reservoir in the North Sea [13]. The pockmarks in this area have diameters up to about 250 m and depths up to around 10 m below the surrounding seafloor level [13,14].

The Troll pockmarks were most likely formed by expulsion of methane from decomposing methane hydrates, caused by increasing temperatures at the end of the last glaciation period about 11000 years ago [15]. No free gas has been detected in shallow sediments of the area at the present time; increasing concentrations of dissolved methane with depth have however been measured from approximately 70 m below seafloor (bsf) [15]. Although sporadic gas bubbles may still be emitted, it is believed that the main force keeping these pockmarks from being filled by sediments is the water-current activity in the craters and porewater flux [15,16].

The Troll field is one of the largest accumulations of petroleum discovered in the North Sea [17]. The reservoir consists of sandstones from the Late Jurassic Sognefjord Formation and is located between 1000 and 1300 m bsf [18]. Although no high level flux of hydrocarbons (seepage) has been detected in this area, diffusion from the petroleum reservoir is likely to occur over geological time, supplying the prokaryotic communities in the overlaying surface sediments with organic carbon [19]. A variety of marine hydrocarbon degrading prokaryotes has been described, mainly from the *Alpha*-, and *Gammaproteobacteria*[20,21]. One example is the genus *Alcanivorax* of the *Gammaproteobacteria*, regarded as a main player in aliphatic hydrocarbon degradation in marine environments [20]. Other genera like *Maricaulis* and *Roseovarius* (*Alphaproteobacteria*) and *Marinobacter* (*Gammaproteobacteria*) are capable of using polycyclic aromatic hydrocarbons (PAHs) as carbon sources [22].

Although prokaryotic communities related to active seepage sites are well studied (e.g. hydrocarbon seeps in the Timor Sea [23], an asphalt volcano in the Gulf of Mexico [24] and Coal Oil Point seep sediments [9]), less is known about the prokaryotic communities in sediments influenced by low level flux (seepage) from underlying hydrocarbon reservoirs over geological time.

In this study we have combined analyses of high throughput (454 GS FLX Titanium) sequenced metagenomes with geochemical data to characterize prokaryotic communities in surface sediments from the Troll area. The aim was to characterize the taxonomic distribution and metabolic potential of the communities, both in general and related to possible hydrocarbon degradation. Further, we wanted to find whether there was an increased potential for methane oxidation or other microbial processes that might support the idea of seepage in the pockmark sediments, or if analyses of the prokaryotic communities would agree with the geological analyses indicating no active hydrocarbon seepage from the pockmarks at the present time [15]. We therefore analyzed sediment samples both from four pockmark samples and one sample from the Troll plain. As references regarding thermogenic hydrocarbon influence, we chose two sediment samples from the seabed in the outer part of the Oslofjord (south of Drøbak, Norway). This area is characterized by Precambrian bedrock, formed more than 542 million years ago, and the presence of thermogenic hydrocarbons is therefore unlikely [18].

## Results

**Figure 1:**
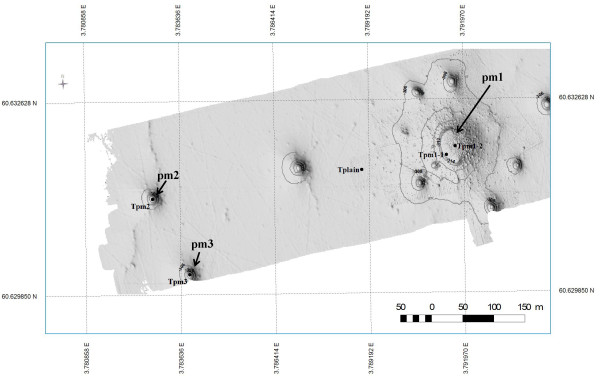
**Map of the Troll sampling sites.** The figure shows the sampling location of the Troll samples. Sample Tplain was taken from the Troll plain. Samples Tpm1-1 and Tpm1-2 were taken from the large pockmark named pm1. Samples Tpm2 and Tpm3 were taken from two smaller pockmarks named pm2 and pm3 respectively.

**Figure 2:**
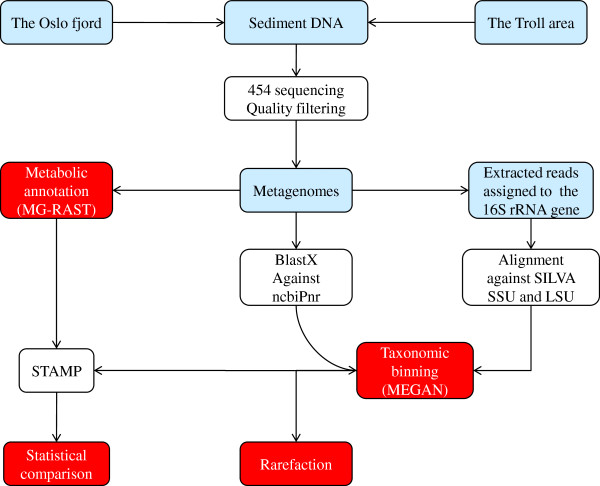
**Flowchart showing the workflow for taxonomic and metabolic binning followed by statistical analyses.** The flowchart gives an overview of the methods used to create and analyze metagenomes from the two sampling areas (The Troll and Oslofjord areas). Abbreviations used in the figure are: MG-RAST (the Metagenomics RAST server), STAMP (Statistical Analysis of Metagenomic Profiles), MEGAN (Metagenome Analyzer), ncbiPnr (NCBI non-redundant Protein Database) and SILVA SSU (small sub unit) and LSU (large sub unit).

The sediment samples from the Troll area were taken from pockmarks (Tpm1-1, Tpm1-2, Tpm2 and Tpm3) as well as one sample from the Troll plain (Tplain) (Figure 1). Sample Tpm1-1 and Tpm1-2 were taken from the same pockmark (named pm1), while samples Tpm2 and Tpm3 were taken from two smaller pockmarks (named pm2 and pm3, respectively). The two Oslofjord samples (OF1 and OF2) were taken from the outer part of the fjord (Additional file 1: Figure S1). Chemical analyses of the sediment porewater, as well as total organic carbon (TOC) and hydrocarbons in the sediments have revealed differences in available carbon and nitrogen sources in the two areas (Table 1 and Additional file 2: Table S1) [25]. Considerably higher concentrations of hydrocarbons (C_10_-C_36_) and a higher ratio of nitrite and nitrate/ammonia, combined with lower concentrations of ammonia and TOC were revealed in the Troll sediments compared to the Oslofjord sediments. To see if these differences were reflected in the prokaryotic communities we used the workflow illustrated in Figure 2.

**Table 1:** Sample site description

**Parameter**	**unit**	**OF1**	**OF2**	**Tplain**	**Tpm1-1**	**Tpm1-2**	**Tpm2**	**Tpm3**
Position	Latitude (N)- longitude (E)	59.594333- 10.633267	59.623800-10.626483	60.631117- 3.787293	60.63132- 3.789782	60.631441- 3.790041	60.630721- 3.78115	60.629635- 3.782211
Water depth	m	212	200	305	315	315	311	311
Sediment depth	cm bsf	5-20	5-20	5-20	5-20	5-20	5-20	5-15
Sediment type		Silty clay	Silty clay	Silty clay	Silty clay	Silty clay	Silty clay	Silty clay
NH_3_	mM	0.3821	0.2464	0.0021	0.0399	0.0387	0.0667	0.0907
NO_3_ + NO_2_	mM	0.0004	0.0004	0.0106	0.0011	0.0019	0.0031	0.0045
TOC	%	1.39	1.46	1.08	0.54	0.64	0.7	0.67
HCO_3_-C	mM	38.25	32.00	10.33	12.08	10.33	16.17	9.60
Cu	mM	0.01	0.01	0.07	0.03	0.06	0.02	0.15
Sum C10-C36	μg/kg	587	368	1276	4993	2840	4547	4289

The table shows the sampling location and an overview of the chemical data obtained by the Norwegian Geotechnical Institute in the Petrogen project [25].

### Sequencing coverage and taxonomic richness

After quality filtering and removal of artificial replicates the number of reads in our metagenomes ranged from 607557 (Tpm2) to 1227131 (Tpm1-2), with average read lengths between 337 ± 131 (Tpm3) and 378 ± 128 (OF2) bases (Table 2). In the following text all percentages are given as percentage of the total reads, after filtering, in each metagenome.

**Table 2:** Metagenome overview

**Metagenome**	**OF1**	**OF2**	**Tplain**	**Tpm1-1**	**Tpm1-2**	**Tpm2**	**Tpm3**
Total sequence (M bases)	342	347	297	239	425	208	303
Total reads	914076	918989	850039	663131	1227131	607557	898796
Average read length (bases)	374 ± 128	378 ± 128	349 ± 134	361 ± 131	346 ± 131	343 ± 131	337 ± 131
Average GC content (%)	48.9 ± 10.7	47.5 ± 10.9	53.9 ± 10.7	49.9 ± 11.5	50.6 ± 12.0	49.3 ± 11.8	49.8 ± 11.0
EGS Mbp	4.9	4.8	5.1	4.7	5.0	4.6	5.0
Total reads assigned to the 16S rRNA gene^1^	926	914	861	776	1358	671	936
(% of total reads)	0.10	0.10	0.10	0.12	0.11	0.11	0.10
Total tDNA reads^1^	3256	3637	2235	2134	3481	2073	2529
(% of total reads)	0.36	0.40	0.26	0.32	0.28	0.34	0.28

^1^ the 16S rRNA gene and tDNA were identified by the WebMGA pipeline.

The table shows general read-based information for the metagenomes.

Rarefaction curves for the most detailed taxonomic level in MEGAN (including all taxa) were leveling off from a straight line at 10% of the metagenome size, indicating that the most abundant taxa were accounted for (Additional file 3: Figure S2). From 1259 (Tpm2) to 1619 (Tpm1-2) taxa were detected in each metagenome at this level. At the genus level the rarefaction curves almost leveled out with 729 (Tpm1-1) to 808 (Tpm1-2) taxa detected, indicating good coverage of the taxonomic richness.

Estimated genome sizes (EGS) for the seven samples were all in the same range and varied between 4.6 (Tpm2) and 5.1 (Tplain) Mbp (Table 2). The fraction of reads assigned to specific genes or functions is therefore assumed to be comparable between the metagenomes. The estimated probability (per read) of sequencing a random gene of 1000 bases was 0.0002 and between 181 and 199 hits could be expected in each metagenome, assuming the gene was present in one copy in all organisms [26]. The most abundant genes of the communities are therefore likely to be accounted for in our metagenomes. Specific genes of interest, present in only small fractions of the community, could however still be missed by chance.

We also analyzed the taxonomy based on extracted reads assigned to the 16S rRNA gene to see if these results were consistent with the results obtained by the complete metagenomes. The number of reads assigned to the 16S rRNA gene ranged from 658 (Tpm2) to 1288 (Tpm1-2), accounting for approximately 0.1% of the reads (Table 2). As expected, rarefaction curves based on these reads were still increasing steeply at the genus level, where only 80 (Tpm2) to 130 (Tpm1-2) taxa were detected (results not shown). Unless otherwise specified, the taxonomic results discussed in the following text are based on total reads.

### Geochemical, taxonomic and metabolic clustering

Due to the complexity of the metagenomes and geochemical data, we performed an exploratory principal component analysis (PCA) to get an overview of the clustering of the samples and parameters tending to co-occur. The ordination analysis was based on the metagenomic data (taxonomic binning at the phylum level and metabolic annotation at level I SEED subsystems). The geochemical data was then fitted onto the ordination using the envfit function of the vegan library in R. The squared correlation coefficient (r^2^) showed that all geochemical parameters with p-values ≤ 0.1 had a high goodness of fit (Additional file 4: Table S2).

**Figure 3:**
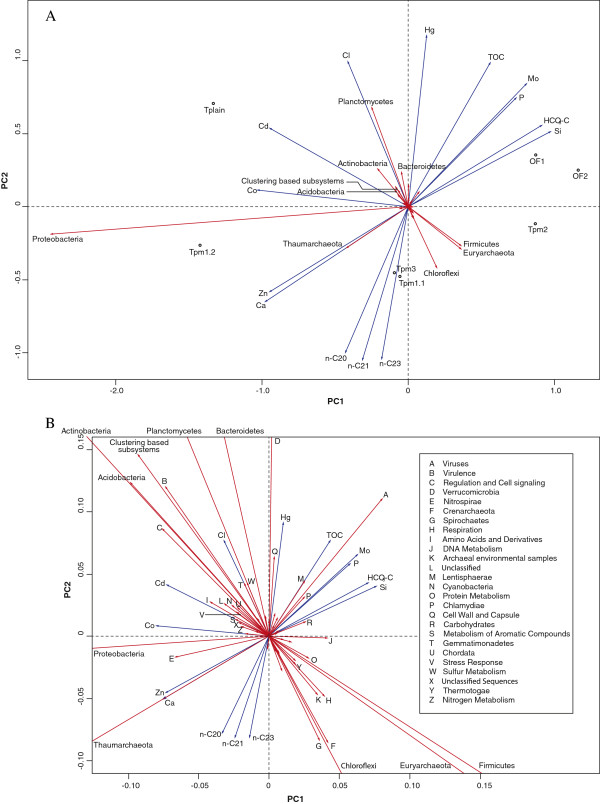
**PCA plot showing the clustering of the samples.** The figure shows a PCA plot based on taxonomic (phylum level) and metabolic (SEED subsystems, level I) parameters combined. The geochemical [25] parameters were overlain using the envfit function of the vegan library in R. The first principal components accounted for 95 % of the variation in the dataset, while the second principal component accounted for 3 %. All metagenome data were given as percent of total reads. The geochemical parameters were normalized by dividing with the standard deviation and subtracting the smallest number from all numbers in each row. Plot **A**: the metagenomic parameters are represented by red arrows. Labels are shown for parameters with Euclidian distance over 0.1 from origin. The geochemical parameters are represented by blue arrows. Only the most significant geochemical parameters are shown (p-value < 0.1). Plot **B**: is an excerpt of plot A, magnifying the central region of the plot. Labels for all metagenomic parameters with Euclidian distance over 0.02 are included.

The PCA plot shows that the two Oslofjord samples (OF1 and OF2) were highly similar and positioned in the top right quadrant (Figure 3A). All the Troll pockmark samples were positioned in the bottom half of the plot. Tpm1-1, Tpm1-2 and Tpm3 were placed in the bottom left quadrant, diagonally opposed to the Oslofjord samples, while Tpm2 was positioned in the bottom right quadrant. Tplain was positioned in the top left quadrant.

The first principal component (PC1) accounted for 95% of the variance in the dataset. Along the PC1 axis Tpm2 was the Troll sample most similar to the Oslofjord samples, while Tplain and Tpm1-2 were positioned furthest away. Tpm3 and Tpm1-1 were placed at an intermediate position.

The abundance of *Proteobacteria* was the most important parameter for the positioning of sites along PC1. *Proteobacteria*, as well as *Thaumarchaeota*, *Planctomycetes* and *Actinobacteria* had high negative scores along this axis. The analysis thereby indicated relatively high abundances of these taxa at the sites placed on the left side of the plot, especially Tpm1-2 and Tplain (Figure 3, Additional file 5: Table S3). *Firmicutes*, *Euryarchaeota*, *Chloroflexi* and Viruses all had high positive scores along PC1 indicating that the samples placed in the right section of the PCA plot (OF1, OF2 and Tpm2) had relatively high abundances of these taxa compared to the other sites.

Although Tpm2 grouped with the Oslofjord samples along PC1, it was separated from the Oslofjord samples by PC2. While *Chloroflexi*, *Euryarchaeota*, *Thaumarchaeota* and *Firmicutes* had high negative scores along PC2, *Bacteroidetes*, *Actinobacteria* and *Planctomycetes* had the highest positive scores along this axis and can therefore be considered as important parameters for the placement of the Oslofjord samples and Tplain in the top half of the plot.

Concerning the carbon sources, the geochemical parameters supported a positive correlation between hydrocarbons (< n-C32) and the Troll samples, while concentrations of bicarbonate and TOC were positively correlated with the Oslofjord samples (Figure 3, Additional file 4: Table S2 and Additional file 6: Figure S3). This correlated well with the metagenomic parameters, where level I SEED subsystem “Carbohydrates” (involved in the metabolism of carbohydrates) was placed in the same quadrant as the Oslofjord samples, while “Metabolism of Aromatic Compounds” (which includes metabolism of aromatic hydrocarbons) was positively correlated to four of the Troll samples, particularly Tplain and Tpm1-2 (Figure 3B).

### Taxonomic classification

The relative representation of the domains in the metagenomes was supported by the 16S rRNA gene data (Additional file 7: Table S4). Consistency between the taxonomy based on all reads and reads assigned to the 16S rRNA gene was also detected at the phylum level (Additional file 8: Figure S4 and Additional file 9: Figure S5 respectively).

### The oslofjord metagenomes

The PCA analysis (Figure 3A) clustered the two Oslofjord metagenomes (OF1 and OF2) together. Statistical comparison of the two metagenomes in STAMP confirmed that they were highly similar. No significant differences in abundance for taxa at either the phylum or the class level were detected. At the genus level only the low abundant genus *Rickettsiella* (OF1: 0.0004%, OF2: 0.0009%), containing intracellular pathogens of arthropods [27], were identified as overrepresented in OF2 compared to OF1. The high similarity of the two Oslofjord metagenomes made them suitable as an out-group for taxonomic comparison against the Troll metagenomes.

### Taxonomic comparison of the troll and oslofjord metagenomes

The genus level was chosen for the taxonomic comparison in STAMP. This level is resolved enough to give a general indication of function and our rarefaction curves indicated good coverage at this level (Additional file 3: Figure S2). Each metagenome from the Troll area was compared to both metagenomes from the Oslofjord. By using a strict significance cut off (including ratio of proportions (RP) ≥ 2), we wanted to identify the differences most likely to be of biological relevance [28]. The analysis identified 196 genera over- or underrepresented in one or more Troll metagenomes compared to the Oslofjord metagenomes (Additional file 10: Table S5). Although differences relative to the Oslofjord metagenomes were detected in all metagenomes from the Troll area (Table 3), no genera were significantly overrepresented in all Troll metagenomes (Additional file 10: Table S5). Only two genera, *Gluconacetobacter* (containing nitrogen-fixing acetic acid bacteria) of the class *Alphaproteobacteria* and *Psychroflexus* (aerobic chemoheterotrophs) of the phylum *Bacteroidetes*, were significantly underrepresented in all Troll metagenomes compared to the Oslofjord metagenomes [29,30].

**Table 3:** Taxa and subsystems differing significantly in abundance

**Samples**	**Genera**		**SEED subsystems**	
	**All taxa**	**Abundant taxa**	**Level I**	**Level III**
**OF1 vs. OF2**	1	0	0	2
**Tplain vs. OF1 and OF2**	141	13	1	60
**Tpm1-1 vs. OF1 and OF2**	23	4	0	3
**Tpm1-2 vs. OF1 and OF2**	124	17	0	52
**Tpm2 vs. OF1 and OF2**	11	4	0	4
**Tpm3 vs. OF1 and OF2**	14	0	1	4

Number of taxa at the genus level and SEED subsystems level I and III with significantly different abundance, based on statistical analyses using STAMP. Abundant taxa are defined as taxa comprising ≥ 0.1 % of all assigned reads in one or more metagenomes.

Most taxa differing significantly in abundance from the Oslofjord metagenomes were detected in Tplain and Tpm1-2 (Table 3). Genera of the phylum *Proteobacteria* (especially the classes *Alphaproteobacteria* and *Gammaproteobacteria*), as well as genera of the archaeal phylum *Thaumarchaeota*, were most frequently overrepresented in these metagenomes, while genera sorting under the bacterial phylum *Firmicutes* and the archaeal phyla *Euryarchaeota* and *Crenarchaeota* were most frequently underrepresented compared to the Oslofjord metagenomes (Additional file 10: Table S5). These trends were also supported by the PCA plot (Figure 3A).

### Abundant taxa at the genus level

**Figure 4:**
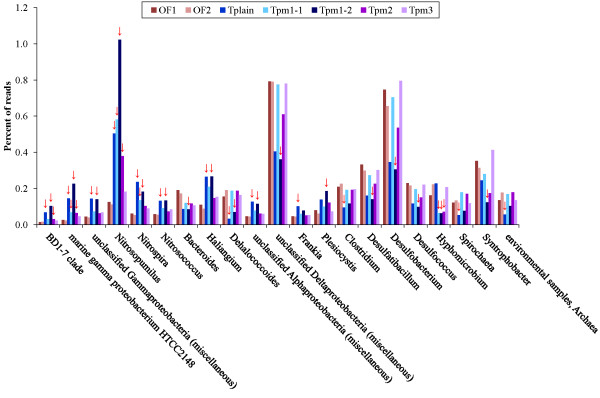
**Significant differences in prokaryote taxonomy between Troll and Oslofjord metagenomes.** The figure shows abundant taxa at the genus level (≥ 0.1 % of the reads in one or more metagenomes) that were classified as significantly different in at least one Troll metagenome compared to both Oslofjord metagenomes in the STAMP analysis. Troll metagenomes significantly different from the Oslofjord metagenomes are marked by red arrows.

We were primarily interested in studying differences among the abundant taxa at the genus level (abundant taxa defined in this study as taxa with more than 0.1% of the reads assigned in one or more metagenomes), since these taxa are likely to have a higher influence on the biochemical activities at the different sites. Altogether 48 abundant bacterial and archaeal taxa were identified at the genus level in the seven metagenomes (Additional file 11: Table S6). Significant differences between one or more Troll metagenomes compared to both Oslofjord metagenomes were detected among 21 of these in the STAMP analysis (Figure 4). Of these 13 were detected in Tplain and 17 in Tpm1-2, respectively (Table 3). Nine genera were detected in both Tplain and Tpm1-2 (Figure 4).

Interestingly, both autotrophic nitrifying genera (*Nitrosopumilus*, *Nitrospira* and *Nitrosococcus*) and oligotrophic marine gammaproteobacteria (OMG: BD1-7, marine gamma proteobacterium HTCC2148 and “unclassified Gammaproteobacteria (miscellaneous)”) were overrepresented in all Troll metagenomes, although not significantly in all, compared to the Oslofjord metagenomes (Figure 4).

### Methanotrophic genera

**Figure 5:**
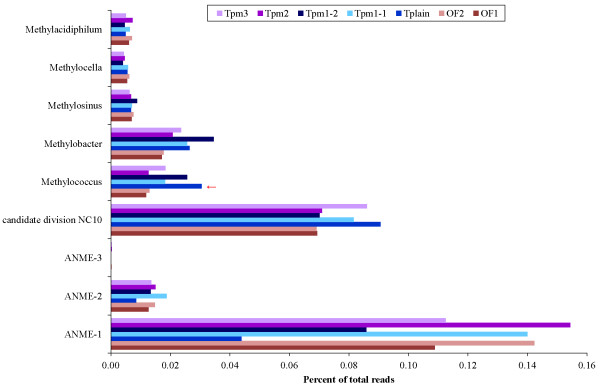
**Potential methanotrophic genera detected.** The figure shows potential methanotrophic taxa detected at the genus level. Genera where Troll metagenomes were significantly different from the Oslofjord metagenomes are marked by red arrows. A subset of reads assigned to the taxon “environmental samples, Archaea” (Significantly underrepresented in Tplain compared to the Oslofjord), further classified as ANME (anaerobic methanotrophic archaea,) are also included.

To see if the sediments from the Troll pockmarks had an increased potential for methane oxidation we searched the metagenomes for known methanotrophic taxa. ANME is not recognized as an independent taxon in the NCBI taxonomy, but an inspection of the reads assigned to “environmental samples, Archaea” showed that these were further assigned to ANME fosmids isolated from Eel River [10] or to “uncultured archaeon”. Inspection of the best hits for the reads assigned to “uncultured archaeon” and reads not assigned beyond the “environmental samples, Archaea” revealed that most of these reads also could be assigned to ANME [10,31,32]. ANME, especially ANME-1, were the most abundant methanotrophs in all metagenomes, except in Tplain, where reads assigned to “candidate division NC10” (assumed to use an “intra-aerobic” methane oxidation pathway [33]) were most abundant (Figure 5).

In the STAMP analysis, only Tplain displayed significant differences in abundance of known methanotrophic genera compared to the Oslofjord metagenomes. The gammaproteobacterial genus *Methylococcus* (aerobic type I methanotrophs) was overrepresented while the abundant taxon “environmental samples, Archaea” was underrepresented in Tplain compared to the Oslofjord metagenomes (Figure 4, Additional file 10: Table S5). Reads assigned to “environmental samples, Archaea” and further to ANME were also two to three times less abundant in Tplain compared to the other Troll metagenomes (Figure 5).

### Metabolic potential

Approximately 12-14% of the reads in each metagenome were assigned to SEED subsystems by MG-RAST (version 2.0) (Additional file 12: Table S7). “Clustering-based subsystems” followed by “Carbohydrates” and “Amino Acids and Derivates”, were the most abundant level I subsystems in all seven metagenomes.

The two Oslofjord metagenomes were highly similar and no significant differences could be detected at SEED subsystem level I in the STAMP analysis. On level III, only two subsystems (“RNA polymerase archaeal initiation factors” and “rRNA modification Haloferax”) were significantly overrepresented in OF2 compared to OF1.

### Metabolic comparison of the Troll and Oslofjord metagenomes

Very few significant differences were detected between the Troll and the Oslofjord metagenomes at SEED subsystems level I in the STAMP analysis. The only significant differences at this level were overrepresentation of the subsystem “Macromolecular Synthesis” in Tplain and underrepresentation of “Prophage” in Tpm3 compared to the Oslofjord metagenomes (Additional file 12: Table S7). At level III however, 79 subsystems were significantly over- or underrepresented in one or more Troll metagenomes compared to the Oslofjord metagenomes (Additional file 13: Table S8). Only one of these (“Archaeal Flagellum”) was significantly underrepresented in all Troll metagenomes compared to the Oslofjord metagenomes.

**Figure 6:**
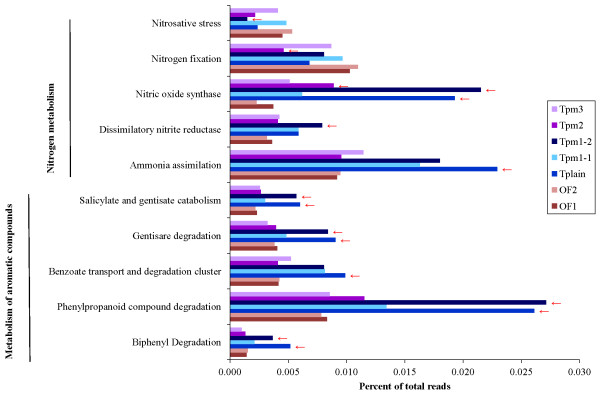
**Significant differences in potential for nitrogen and aromatic compound metabolism between Troll and Oslofjord metagenomes.** The figure shows differences in level III SEED subsystems involved in metabolism of nitrogen and aromatic compounds where at least one Troll metagenomes was significantly different from both Oslofjord metagenomes in the STAMP analysis. Troll metagenomes significantly different from the Oslofjord metagenomes are marked by red arrows.

Concerning petroleum degradation, several subsystems involved in metabolism of aromatic hydrocarbons were among those significantly overrepresented in Tplain and Tpm1-2 compared to the Oslofjord metagenomes (Additional file 13: Table S8). These subsystems (except “Benzoate transport and degradation cluster”) were also considerably more abundant in Tplain and Tpm1-2 than in the other Troll metagenomes (Figure 6). This was also seen in the PCA analysis, where the level I SEED subsystem “Metabolism of Aromatic Compounds” was contributing to the separation of Tplain and Tpm1-2 from the Oslofjord samples (Figure 3).

**Figure 7:**
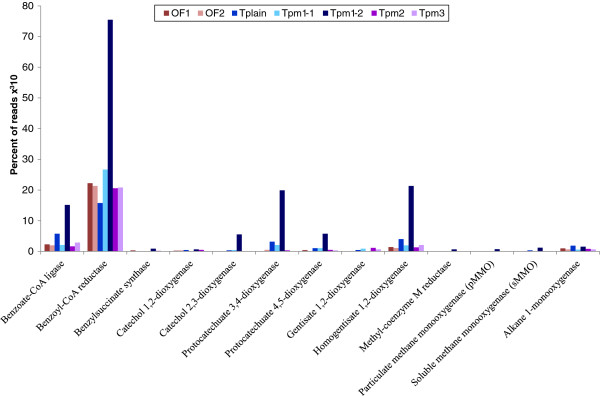
**Key genes for hydrocarbon degradation detected.** The figure shows reads assigned to a selection of key genes for hydrocarbon degradation detected in the metagenomes. The reads were identified by search in MG- rast 3; and against a reference library for alkane monooxygenase.

Identification of selected key enzymes for hydrocarbon degradation further supported a higher potential for hydrocarbon degradation in Tplain and Tpm1-2 compared to the other samples (Figure 7). Anaerobic degradation of several aromatic compounds is often funneled through benzoate and benzoyl-CoA by benzoate-CoA ligase and subsequent dearomatization by benzoyl-CoA reductase [34]. The anaerobic activation step of toluene and several other aromatic hydrocarbons with fumarate addition can be catalyzed by benzylsuccinate synthase. We searched for these anaerobic key enzymes as well as for several dioxygenases involved in aerobic ring-cleavage of the aromatic intermediates catechol, protocatechuate, gentisate and homogentisate.

Both benzoate-CoA ligase, and several dioxygenases (e.g. protocatechuate 3,4-dioxygenase and homogentisate 1,2-dioxygenase) were overrepresented in the metagenomes from Tplain and Tpm1-2. Alkane 1-monooxygenase (*alk*B), the key enzyme in alkane degradation, was also seen to be more abundant in Tplain and Tpm1-2 than in the other metagenomes. A few reads assigned to the key genes in anaerobic (methyl-coenzyme M reductase) and aerobic (particulate and soluble methane monooxygenase) methane oxidation were also detected in the Tpm1-2 metagenome. The soluble methane monooxygenase was identified in the metagenomes from Tplain and OF2 as well.

An inspection of the level 3 SEED subsystems sorting under “Nitrogen Metabolism” (Figure 6) revealed that “Ammonia assimilation” was overrepresented in all Troll metagenomes, although the difference was only significant for Tplain. This fits well with the overrepresentation of autotrophic nitrifiers in the Troll metagenomes.

Denitrification represented by “Dissimilatory nitrate reductase” was also overrepresented in the Troll metagenomes (significantly so in Tpm1-2) [35]. Further, “Nitric oxide synthase” was significantly overrepresented in Tplain, Tpm1-2 and Tpm2 compared to the Oslofjord metagenomes. Most reads assigned to this subsystem were classified as putative cytochrome P450 hydroxylase. Cytochrome P450 enzymes are ubiquitous and involved in a broad range of chemical reactions, including aromatic hydrocarbon degradation [36].

In accordance with the taxonomic comparison, Tplain and Tpm1-2 differed most from the Oslofjord metagenomes also in respect of metabolic potential (Table 3).

## Discussion

The PCA analysis separated the Troll samples from the Oslofjord samples (see Figure 3). This supports the Oslofjord metagenomes as a suitable out-group for comparison against the Troll metagenomes. The plotted geochemical parameter fitted well onto the ordination and supported a correlation between available carbon sources and the clustering of the samples. The plot further visualized correlations between geochemical and metagenomic (taxonomic and metabolic) parameters.

To better reflect the situation in the environment, taxonomic and metabolic parameters used in the PCA ordination were given as percent of total reads. This way high abundant taxa and subsystems were given higher influence on the ordination than their low abundant counterparts.

The PCA analysis was based on metagenomic data from the phylum and SEED subsystem I levels. The taxonomic and metabolic classification on this level provides a limited resolution compared to the genus and SEED subsystem III levels used for the in-depth metagenomic analysis. Further, not all metagenomic reads could be assigned; neither was all possible geochemical parameters measured. Still, the exploratory PCA analysis provided a valuable insight into the effects of environmental conditions on community composition and differentiations. The results further supported the more detailed analyses performed in this study.

### Variation in the prokaryotic communities between the two sampling areas

The taxonomic comparison of the Troll and Oslofjord areas showed a general overrepresentation of autotrophic nitrifiers and OMG in the Troll area (see Figure 4). Both *Nitrosopumilus* and OMG are known to thrive in oligotrophic environments and their overrepresentation could therefore be due to lower TOC in the Troll area than in the Oslofjord (see Table 1) [37,38]. An active nitrifying community in the Troll sediments was further supported by a relatively higher nitrite and nitrate to ammonia ratio as well as an increased genetic potential for ammonia assimilation in the Troll sediments compared to the Oslofjord (see Figure 6). Ammonia is however assimilated by other prokaryotes as well, especially in oligotrophic environments [39].

The PCA analysis showed a positive correlation between “Nitrogen metabolism” (Figure 3B) and concentrations of nitrite and nitrate measured in the pore water (Additional file 6: Figure S3). A positive correlation was also indicated between these parameters and the abundance of the phyla *ProteobacteriaNitrospiraeThaumarchaeota* (which harbors the nitrifying genera *NitrosococcusNitrospira* and *Nitrosopumilus* respectively). Although the phylum *Proteobacteria* is highly diverse, the largest fraction of reads assigned to *Nitrospirae* and *Thaumarchaeota* were classified as *Nitrospira* and *Nitrosopumilus* respectively. The PCA analysis thereby supports a positive correlation between the level I subsystem “Nitrogen metabolism”, nitrifiers and elevated concentrations of nitrite and nitrate. The plot further indicated a negative correlation between these parameters and the pore water ammonia concentration. The considerably lower ammonia concentration measured in the Troll samples compared to the Oslofjord samples could be a result of the nitrifiers’ effective metabolism of ammonium. Especially *Nitrosopumilus*, strain SCM1, has been shown to have a high affinity for ammonia [38].

Interestingly, the PCA plot indicated a strong positive correlation between *Thaumarchaeota* (including the genus *Nitrosopumilus*) and the geochemical parameters zinc and calcium.

The correlation between calcium and *Thaumarchaeota* could in part be explained by the calcium carbonate mound found close to Tpm1-2, where the *Thaumarchaeota* were most abundant.

### High variance detected within the Troll area

The high variance present among the Troll samples indicates environmental differences related to the different structures (e.g. pockmarks and carbonate structures) on the seabed in the area (see Figure 1). Interestingly the Tpm1-1 and Tpm1-2 samples (both taken from pm1) were dissimilar, possibly due to the pockmark’s large size and heterogeneity. Close to the eastern slope, where sample Tpm1-2 was taken, biogenic carbonate structures probably formed during previous methane seepage could be seen (data not shown) [16]. Meanwhile, no such carbonate structures were detected at the western slope where sample Tpm1-1 was taken.

The PCA analysis placed Tplain and Tpm1-2 considerably further left along PC1 than the other Troll samples (Figure 3). The most striking difference in geochemical composition between Tplain and Tpm1-2 on one side and Tpm1-1, Tpm2 and Tpm3 on the other was the considerably lower concentration of aliphatic hydrocarbons in Tplain and Tpm1-2 compared to the other Troll samples (see Table 1). This trend was also seen in the PCA plot (Figure 3 and Additional file 6: Figure S3).

In combination with a higher taxonomic and metabolic potential for hydrocarbon degradation, this indicates a more active hydrocarbonoclastic subcommunity in Tplain and Tpm1-2. Although subsystems involved in degradation of aromatic hydrocarbons were detected in all metagenomes, significant overrepresentation compared to the Oslofjord metagenomes could only be detected in Tplain and Tpm1-2; thereby supporting a more active hydrocarbon degrading community in these samples (see Figure 6). The Tplain and especially the Tpm1-2 metagenomes also had a higher fraction of reads assigned to key genes for hydrocarbon degradation than the other samples (see Figure 7). Further, known hydrocarbon degrading genera from both *Alphaproteobacteria* (like *Sphingomonas* and *Roseovarius*) and *Gammaproteobacteria* (like *MarinobacterColwellia* and *Alcanivorax*) were overrepresented in Tplain and Tpm1-2 compared to the Oslofjord metagenomes (Additional file 10: Table S5) [20,22,40,41]. This trend can also be seen in the PCA plot where the parameters *Proteobacteria* (containing most of the known hydrocarbon degraders) and “Metabolism of Aromatic Compounds” (containing subsystems for degradation of aromatic hydrocarbons) are important contributors in separating Tplain and Tpm1-2 from the other samples.

In general aromatic hydrocarbons are more recalcitrant than aliphatic hydrocarbons to microbial degradation [42]. The Troll samples all share the common predominant source of hydrocarbons, the underlying oil and gas reservoir. The increased genetic potential for degradation of aromatic hydrocarbons in Tplain and Tpm1-2 is therefore likely to be a result of sequential degradation of the various fractions in oil. A more active hydrocarbonoclastic subcommunity in Tplain and Tpm1-2 could have degraded a larger fraction of the less recalcitrant aliphates, forcing a shift in the metabolism towards increased degradation of aromatic hydrocarbons at the sampling time.

The seabed is a dynamic environment, and a theory by Hovland and coworkers proposes that as old pockmarks are closed down new ones are created as a result of changes in fluid flow pathways over time [16]. Higher potential for hydrocarbon degradation, possibly related to a more active hydrocarbonoclastic subcommunity in Tplain and Tpm1-2, could be explained by increased bioavailability of essential nutrients (e.g. nitrogen and phosphorous) and metals involved in hydrocarbon degradation at these sites compared to the other Troll sites, as a result of increased porewater seepage. Increased porewater seepage could also bring about a slightly higher hydrocarbon availability, especially of the more aqueous soluble hydrocarbons, which could sustain a more active hydrocarbonoclastic subcommunity at Tplain and Tpm1-2 [23].

At Tpm1-2 a potential increase in porewater seepage could be explained by the carbonate mound identified close to the sampling site. This carbonate mound could constitute a seal for gas migrating towards the seafloor, thereby increasing the pressure in the porewater forced out along its sides [16].

Further, differences in exposure to water-current activity could also affect the bioavilibility of nutrients and community structure. Previous investigation of fauna in large Troll pockmarks has indicated the possibility for increased currents or turbulence at the eastern slope of the pockmarks in the area [14]. Likewise, there is no protection from the water current on the Troll plain.

### Methane oxidation in pockmark sediments

Although methanotrophs contributed to all seven metagenomes, no general overabundance could be detected in the Troll pockmark metagenomes compared to the Oslofjord metagenomes, supporting the geochemical conclusion that there is no, or very low, active methane seepage in these pockmarks at the present time [15]. We did recognize marker genes for aerobic methane oxidation in Tpm1-2 and Tplain. This could be related to the slight overabundance of aerobic methanotrophic taxa (e.g. *Methylococcus*) in these samples. Interestingly, reads associated with ANME were two to three times less abundant in the metagenome from the Troll plain (Tplain), than in the Troll pockmark metagenomes (Tpm1-1, Tpm1-2, Tpm2 and Tpm3) where ANME accounted for up to 0.17% of the reads. ANME are less abundant in the Troll pockmarks than in active, methane-seeping pockmarks like Gullfaks, Tommeliten and Nyegga, where ANME sequences dominated the archaeal 16S libraries in surface sediments [6,43]. In contrast, aerobic ammonia oxidizing *Nitrosopumilus* was clearly the most abundant archaeal genus in the Troll metagenomes. *Nitrosopumilus* and other Marine Archaeal Group I representatives have also previously been detected in the outskirts of hydrocarbon seepages, methane-hydrate sediments, oil spills and hydrothermal vents [41,44-47]. Recently Marine Archaeal Group I representatives were also identified as the dominating archaea in surface sediments (0–3 cm bsf) overlaying the zone of anaerobic methane oxidation (AOM) in sediments of an active methane seeping pockmark [48].

Since the zone for AOM is deeper in sediments with low level diffusion based seepage, compared to sediments with active methane seepage [45], we can not exclude that AOM might be more important in deeper layers of the sediments. CO_2_ produced by anaerobic oxidation of methane [12] (or anaerobic degradation of other hydrocarbons ascending from the reservoir [19,49]) in deeper layers of the Troll sediments would provide an additional carbon source for *Nitrosopumilus*, and other predominantly autotrophic nitrifiers, generally overrepresented in the oligotrophic Troll sediments. The predominantly autotrophic nitrifiers overrepresented in these oligotrophic sediments might therefore have a function in turning CO_2_, in part originating from hydrocarbons, back into organic carbon and thereby reducing the emission of this greenhouse gas to the seawater. The nitrifiers could further play a role providing terminal electron acceptors for nitrate reducing hydrocarbon degraders (often found whiten the *Betaproteobacteria*[50,51]).

We did not find significantly overrepresented subsystems related to CO_2_ fixing pathways in our analysis. This could in part be related to difficulties in assigning metagenomic reads to function. *Nitrosopumilus,* the most abundant of the nitrifiers overrepresented in the Troll area, is assumed to use a variant of the 3-hydroxypropionate/4-hydroxybutyrate pathway (3HP/4HB) for CO_2_ fixation [52]. This pathway is not well defined in the SEED subsystems of MG-rast (version 2). Further, although *N. maritimus* most likely uses the same reaction sequences as described for *Metallosphaera sedula*, not all reactions are catalyzed by identical enzymes [52]. It is still not clear whether ammonia oxidizing archaea are dependent on autotrophy or not. A mixotrophic lifestyle has been indicated for *Nitrosopumilus* and other (mainly marine) group I.1a *Thaumarchaeota*, while heterotrophic growth has been observed for *Thaumarchaeota* of group I.1b (most common in soils) [52-55]. Since 4-hydroxybutyryl-CoA dehydratase/vinylacetyl-CoA-Delta-isomerase, a characteristic key gene of the 3HP/4HB cycle [56], has been detected by the KEGG Automatic Annotation Server (KAAS) [57,58] among metagenomic reads assigned to *N. maritimus* from the Troll metagenomes in a separate study [59] it is likely that *Nitrosopumilus* in the Troll area has the genetic potential for autotrophy.

## Conclusions

Most taxa were present in all metagenomes and differences in community structure and metabolic potential between them were mainly due to abundance variation. Despite detection of a few reads assigned to key enzymes for methane oxidation in Tpm1-2, our analyses revealed no general increase in the potential for methane oxidation in the surface sediments of Troll pockmarks compared to the Oslofjord. The analyses are thereby supporting geological analyses indicating no, or very low, methane seepage at the present time. Despite high concentrations of hydrocarbons in the Troll area, compared to the Oslofjord, significantly increased potential for hydrocarbon degradation could only be detected in two of the Troll metagenomes. Overrepresentation of subsystem and key enzymes supported an increased potential for aromatic hydrocarbon degradation in these samples.

The proposed extended use of aromatic hydrocarbons as a carbon source could be a result of the lower alkane concentrations measured in these samples compared to the other Troll samples. Given the placement of the sampling sites, less bioavailability of nutrients essential for hydrocarbon degradation is a likely factor limiting the hydrocarbonoclastic subcommunities at the other sites.

The most evident difference between the two sampling areas was an overabundance of predominantly autotrophic nitrifiers, especially *Nitrosopumilus*, in the Troll metagenomes compared to the Oslofjord. Given the great depth of the hydrocarbon-containing sediments in the Troll area, substantial sequential anaerobic degradation and oxidation of hydrocarbons is likely to occur. Migration of degradation products, including CO_2_, up through the sediments could provide an additional source of carbon for the nitrifiers thriving in the area. This subcommunity could therefore play an important role turning CO_2_, partially originating from hydrocarbon degradation, back into organic carbon in these dark oligotrophic sediments. The oxidation of ammonia to nitrite and nitrate in this autotrophic process could also boost the supply of terminal electron acceptors for hydrocarbon degradation.

## Methods

### Sampling

The sediment samples from Troll (Tplain, Tpm1-1, Tpm1-2, Tpm2 and Tpm3) were collected in the northern North Sea by the survey vessel Edda Fonn in March 2005. Samples Tpm1-1, Tpm1-2, Tpm2 and Tpm3 were taken from the bottom of three different pockmarks, while sample Tplain was taken from the Troll plain (Figure 1). The samples were collected using a combination of a 0.5 m ROV-operated shallow core device and a ROV manipulator. Details on the sampling locations are listed in Table 1 and Additional file 2: Table S1.

Samples OF1 and OF2 were taken approximately 2 km apart, south of Drøbak in the Oslofjord, Norway. The samples were collected by a big gravity corer with a 110 mm PVC tube mounted with blade and sand trap from a survey with the research vessel FF Trygve Braarud in December 2005.

The core liners were sealed upon arrival at the ship and kept at 4-10 °C during transport to the laboratory. The cores were opened under aseptic conditions and samples for DNA extraction were taken from the core centre to avoid cross contamination from the core liner. Samples from 5–20 cm bsf were used to avoid recent sediments and possible surface contaminations. Sediment from the core centre used for DNA extraction was homogenized before use. Approximately 0.5 to 1 g sediment was needed to extract 1 μg of DNA prior to purification (measured by NanoVue Fisher Scientific). The rest of the core was homogenized and used for geochemical analyses.

### DNA extraction

Total genomic DNA was extracted with a FastDNA®SPIN for Soil Kit (MP Biomedicals) and cleaned using Wizard DNA Clean-Up (Promega) according to the manufacturer’s instructions. The DNA quality was assessed by agarose gel electrophoresis and by optical density using a NanoDrop instrument (NanoDrop Products, Thermo Scientific).

### 454 sequencing

4–20 μg DNA was used for sequencing. Sample preparation and sequencing of the extracted DNA were performed at the High Throughput Sequencing Centre at CEES, University of Oslo [60] according to standard GS FLX Titanium protocols.

The samples were tagged, mixed and sequenced on a 70x75 format PicoTiterPlate^TM^ on a GS FLX titanium instrument. Each sample was run twice, generating two datasets with different read length distributions for each sample. Since the datasets from each sample had very similar GC content distribution, all available sequence data for each sample was pooled.

The metagenomic reads have been submitted to the Genbank Sequence Read archive [GenBank: SRP009243].

### Quality filtering

The complete datasets were analyzed with Prinseq to determine the sequences quality scores [61]. For each sample we performed quality filtering to remove low quality reads (reads containing ≥ 10 ambiguous bases, or homopolymers of ≥ 10 bases) using mothur [62]. Exact duplicates were removed from the remaining reads using an in-house script. Artificial replicates were removed using cdhit-454 with standard settings except minimal identity, which was set to 98% [63].

### Effective Genome size (EGS) and sampling probability

The effective genome size (EGS) for each metagenome was estimated according to the method developed by Raes et al. [64], using the constants a = 18.26, b = 3650 and c = 0.733. A protein reference database containing the 35 single copy COGs in question were downloaded from STRING (9.0) [64,65]. BlastX was conducted at the freely available Bioportal computer service [66,67].

Sampling probability of a random universal single copy gene (1000 bases) and expected number of reads detected was calculated according to Beszteri et al. [26].

### Taxonomic annotation

The metagenomic reads were taxonomically classified by BlastX against the NCBI non-redundant Protein Database (ncbiP-nr) [67]. The computation was performed at the freely available Bioportal computer service [66]. Maximum expectation-value was set to 10^-3^, maximum 25 alignments were reported per hit.

The BlastX output files were analyzed according to NCBI-taxonomy in the program MEGAN, version 4 [68,69] with default LCA-parameters (Min Score: 35, Top Percent: 10.0 and Min Support: 5). All taxa were enabled.

The metagenomes were also analyzed for the presence of gene fragments encoding ribosomal RNA’s using the rRNA and tRNA prediction tool of the WebMGA pipeline [70,71]. An expectation value cut off of 10^-20^ was used for the predictions. The reads assigned to the 16S rRNA gene were taxonomically classified by BlastN against the SILVA SSU and LSU databases (version 108). An expectation value cut off of 10^-5^ was used in the blast analyses and maximum 25 alignments were reported. The BlastN output files were combined and analyzed in MEGAN version 4 [68,69] using the silva2ncbi mapping file. To better capture the taxonomic richness in the relatively few reads assigned to the 16S rRNA gene we lowered the min support threshold while the min score threshold was increased to insure good quality of the hits (LCA parameters: min Score: 50, top percent 10 and min support 1).

### Metabolic annotation

The metagenome reads were assigned to SEED subsystems on the MG-RAST server (version 2.0) [72,73]. Maximum expectation-value was set to 10^-5^, minimum alignment length was set to 100 bases. The SEED subsystems at MG-RAST are organized in a hierarchical structure with three levels, which in the remaining text are referred to as levels I, II, and III, where level III is most detailed.

We also searched the metagenomes for key genes involved in hydrocarbon degradation at MG-RAST (version 3.1.2). Maximum expectation-value was set to 10^-5^, minimum alignment length was set to 50 bases. The genes for the following enzymes where searched; Benzoate-CoA ligase (EC 6.2.1.25), benzoate CoA reductase (EC1.3.99.15) (subunits BadD, E, F, G) benzylsuccinate synthase (EC 4.1.99.11), catechol 1,2-dioxygenase (EC 1.13.11.1), catechol 3,4-dioxygenase (EC 1.13.11.2), protocatechuate 3,4-dioxygense (EC 1.13.11.3) (alpha and beta), gentisate 1,2-dioxygenase (EC 1.13.11.4), homogentisate 1,2-dioxygenase (EC 1.13.11.5), protocatechuate 4,5-dioxygenase (EC 1.13.11.8) (alpha and beta), methyl-coenzyme M reductase (EC 2.8.4.1) (alpha), methane monooxygenase (EC 1.14.13.25) (particulate: pMMO and soluble: sMMO).

The metagenome reads were further compared to a protein sequence library for alkane monooxygenase (*alk*B) on the freely available Bioportal computer service [66]. The reference library for *alk*B was downloaded from Fungene (Functional gene pipeline & repository) version v6.1 [74], including only sequences with a score (bits saved) of 100 or more from the HMMER Hidden Markov Model search against NCBIs non-redundant protein database. We used blastX against the protein sequences of the enzyme library with a maximum expectation value of 10^-20^[67]. Maximum one alignment was reported.

### PCA analysis

The PCA-plots were created using the vegan library in R [75-77]. The ordination was based on reads assigned at the phylum level in MEGAN version 4 (“Not assigned” and “No hits” were excluded) and to level I SEED subsystems extracted from MG-RAST (“No hits” was excluded) [68,69]. All metagenome data were given as percent of total reads. Symmetric scaling, for both parameters and sites, was used in the plot. The geochemical parameters [25] were fitted onto the ordination using the envfit function. The lengths of arrows for the fitted parameters were automatically adjusted to the physical size of the plot, and can therefore not be compared across plots. To account for the different measuring units, all geochemical parameters were normalized by dividing with the standard deviation and subtracting the smallest number from all numbers in each row.

### Rarefaction analysis

Rarefaction analysis was performed in MEGAN version 4 [68,69]. The MEGAN program uses an LCA-algorithm to bin reads to taxa based on their blast-hits. This results in a rooted tree. The leaves in this tree are then used as OTUs in the rarefaction analysis. The program randomly chooses 10%, 20% … 100% of the total number of reads as subsets. For each of these random subsets the number of leaves (hit with at least 5 reads (min-support)) was determined. This sub sampling is repeated 20 times for each percentage and then the average value is used for each percentage.

The analysis was done for all taxa (including *Bacteria*, *Archaea*, *Eukaryota*, viruses and unclassified sequences) at the genus level, and at the most detailed level (typically species or strain) of the NCBI taxonomy in MEGAN.

### Comparison of the metagenomes

Comparison tables of absolute numbers for different bacterial and archaeal taxonomic (NCBI) levels for the seven metagenomes were extracted from MEGAN [68,69]. Likewise, comparison tables of absolute numbers of reads assigned to SEED subsystems in the seven metagenomes were extracted from MG-RAST [72,73].

Statistical analyses were done in STAMP (Statistical Analysis of Metagenomic Profiles) (version 1.07) [28]. The following settings were used: Parent level; Entire sample (all reads), Statistical test; Fishers exact test (two sided), CI-method; Asymptotic (0.95%), Multiple test correction; Story FDR (For the comparison of metabolic potential Benjamini-Hochberg FDR was used to ensure a uniform distribution of p-values). The following settings were used for filtering significant results: q-value filter; 0.05, minimum sequences from each sample; 6, effect size filter; ratio of proportions (RP) ≥ 2.00).

The two metagenomes from the Oslofjord (OF1 and OF2) were compared at the phylum, class, genus and species level, as well as SEED subsystem levels I and III.

To identify differences between the two sampling areas the individual Troll metagenomes (Tplain, Tpm1-1, Tpm1-2, Tpm2 and Tpm3) were compared to both Oslofjord metagenomes (OF1 and OF2) at the genus level and SEED subsystem levels I and III. Difference in abundance had to be detected compared to both Oslofjord metagenomes to be considered.

Taxa at the genus level with ≥ 0.1% of the reads were defined as abundant.

### Geochemical analyses

The geochemical data were obtained by the Norwegian Geochemical Institute (NGI) in the Petrogen project [25]. The method is described in Additional file 14: Methods for geochemical data.

## Authors’ contributions

OEH carried out the taxonomic, metabolic and statistical analyses, calculated EGS and drafted the manuscript. THAH carried out the quality filtering, initial taxonomic blasting and annotation of the reads assigned to the 16S rRNA gene. OEH and THAH isolated DNA from the sediment samples. AGR conceived the study, participated in its design, acquired the sediment samples and conducted the marker gene search on MG-RAST 3. OEH, THAH, TK and KSJ participated in the design of the study. All authors revised and approved the final manuscript.

## Supplementary Material

Additional file 1 **Figure S1. Sampling site locations.** A) The figure shows a map where the Troll and Oslofjord sampling sites are marked by yellow pins. B) Detailed map of the Oslofjord sampling sites. Click here for file

Additional file 2 **Table S1. Sample site description and chemical data.** The table shows details on sampling location and chemical data obtained by the Norwegian Geotechnical Institute in the Petrogen project [25]. Click here for file

Additional file 3 **Figure S2. Rarefaction curves created in MEGAN.** Rarefaction analysis was performed at the most resolved and genus level of the NCBI taxonomy in MEGAN for each metagenome. The curves included all taxa (*Bacteria*, *Archaea*, *Eukaryota*, viruses and unclassified sequences). The solid lines represent leaves at the most detailed taxonomic level, while the stippled lines represent leaves at the genus level. Click here for file

Additional file 4 **Table S2. Score table for the geochemical parameters.** The table shows the scores of the geochemical parameters fitted onto the PCA ordination shown in Figure 3. The first two columns gives the direction cosines of the vectors, r^2^ gives the squared correlation coefficient. The parameters are sorted by increasing p-values. Click here for file

Additional file 5 **Table S3. Metagenomic parameter scores.** The table shows metagenomic parameters scores for the first and second principal component in the PCA analysis. Click here for file

Additional file 6 **Figure S3. PCA plot showing all measured geochemical parameters.** The figure shows the same PCA plot as Figure 3, but displays all the measured geochemical parameters labeled by numbers. Click here for file

Additional file 7 **Table S4. Reads assigned at the domain level in MEGAN.** Numbers are given as percent of total reads (numbers based on the reads assigned to the 16S rRNA gene). Click here for file

Additional file 8 **Figure S4. Taxonomic distribution of prokaryotes based on all reads at the phylum level.** The figure shows the taxonomic distribution of prokaryotes in the metagenomes at the phylum level (*Proteobacteria* are presented at the class level) based on MEGAN analysis (Min Score: 35, Top percent: 10 and Min Support: 5) of all reads after blast against NCBIs non redundant Protein database. Click here for file

Additional file 9 **Figure S5. Taxonomic distribution of prokaryotes based on reads assigned to the 16S rRNA gene at the phylum level.** The figure shows the taxonomic distribution of prokaryotes in the metagenomes at the phylum level (*Proteobacteria* are presented at the class level) based on MEGAN analysis (Min Score: 50, Top percent: 10 and Min Support: 1) of reads assigned to the 16S rRNA gene after blast against the SILVA SSU and LSU databases. Click here for file

Additional file 10 **Table S5. Significantly over or underrepresented genera in Troll metagenomes compared to both Oslofjord metagenomes.** Genera differing significantly in one or more Troll metagenomes compared to both Oslofjord metagenomes after statistical analysis in STAMP. Click here for file

Additional file 11 **Table S6. Abundant bacterial and archaeal taxa at the genus level.** Taxa with ≥ 0.1% of the reads in one or more metagenomes are presented. Numbers are given as percent of total reads. Click here for file

Additional file 12 **Table S7. Relative proportion of reads assigned to SEED subsystems (level I).** Abundances are presented as percent of total reads. Subsystems where a Troll metagenome showed significant differences compared to both Oslofjord metagenomes in the STAMP analysis are marked with an asterisk. Click here for file

Additional file 13 **Table S8. Significantly over or underrepresented subsystems (level III) in Troll metagenomes compared to both metagenomes from the Oslofjord.** Level III subsystems differing significantly in one or more Troll metagenomes compared to both Oslofjord metagenomes after statistical analysis in STAMP. Click here for file

Additional file 14 **Methods for geochemical data.** Methods used to obtain geochemical data [25]. Click here for file
