# Supplementary figures and images for: Metagenomic and geochemical characterization of pockmarked sediments overlaying the Troll petroleum reservoir in the North Sea
Source: BMC Microbiol. 2012 Sep 11;12:203. doi: 10.1186/1471-2180-12-203 (PMC3478177; doi:10.1186/1471-2180-12-203)

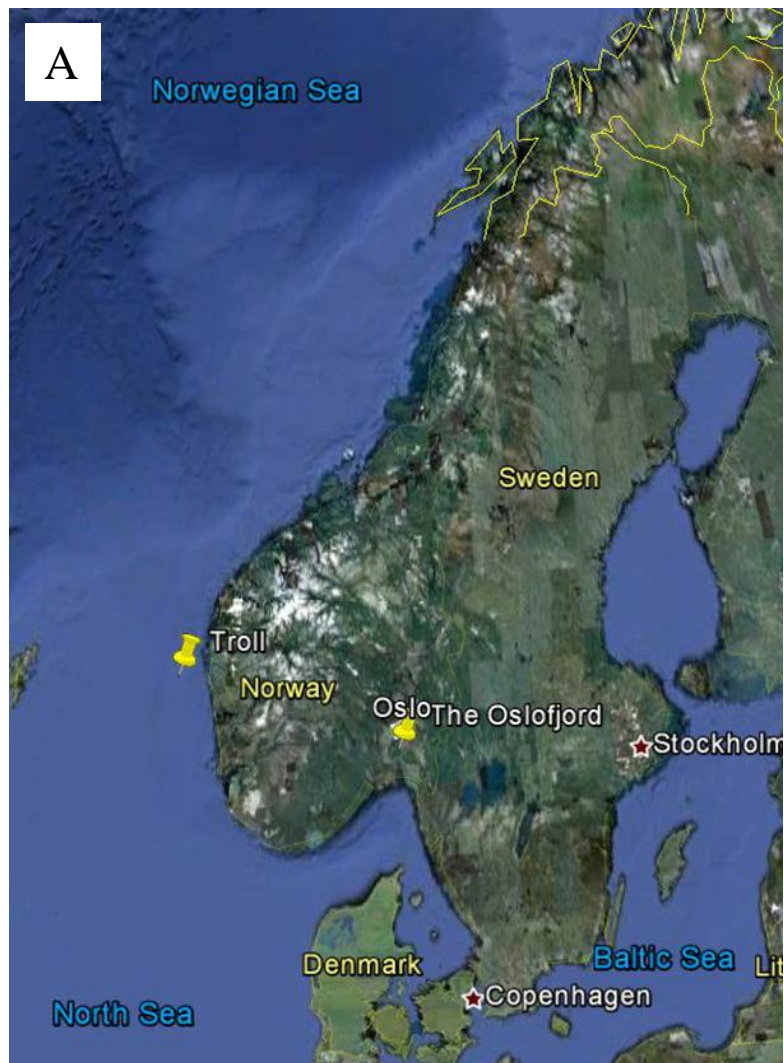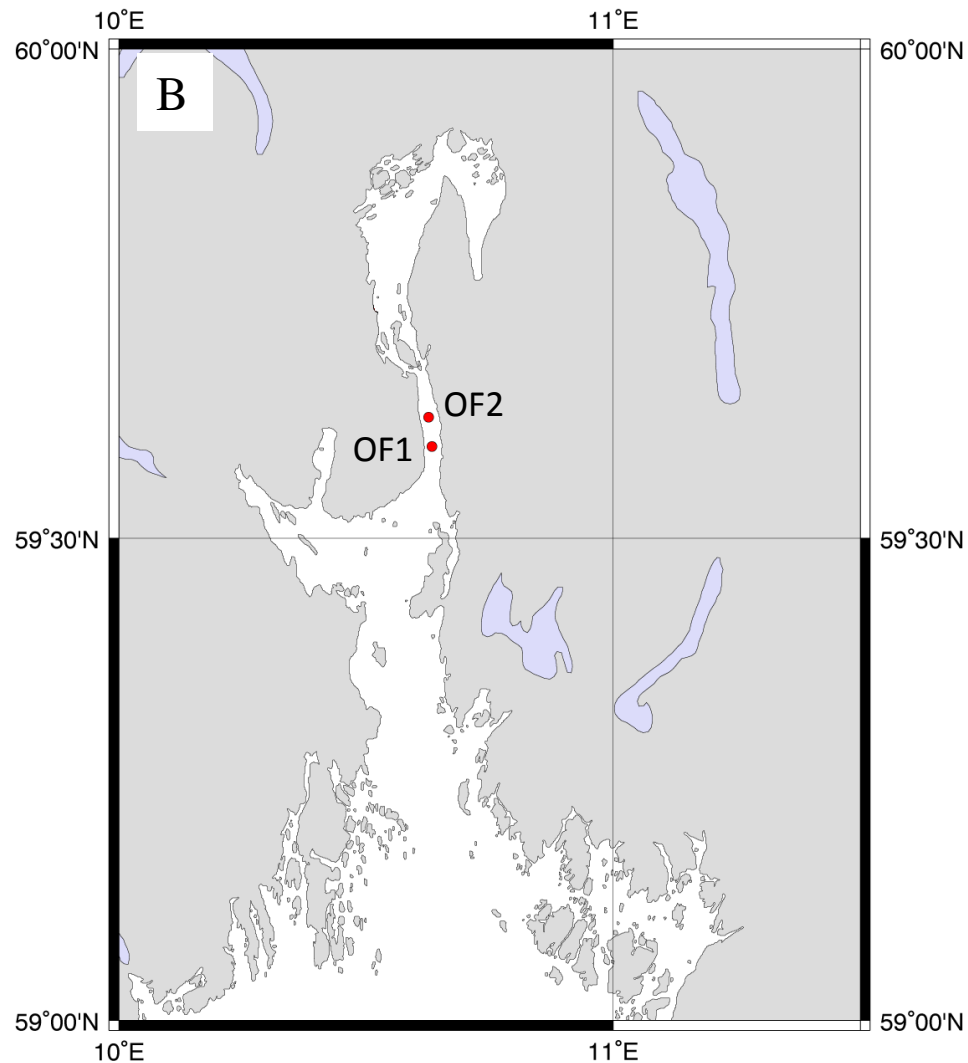

Supplement: Additional file 1 — Figure S1. Sampling site locations. A) The figure shows a map where the Troll and Oslofjord sampling sites are marked by yellow pins. B) Detailed map of the Oslofjord sampling sites. [file 1471-2180-12-203-S1.pdf]

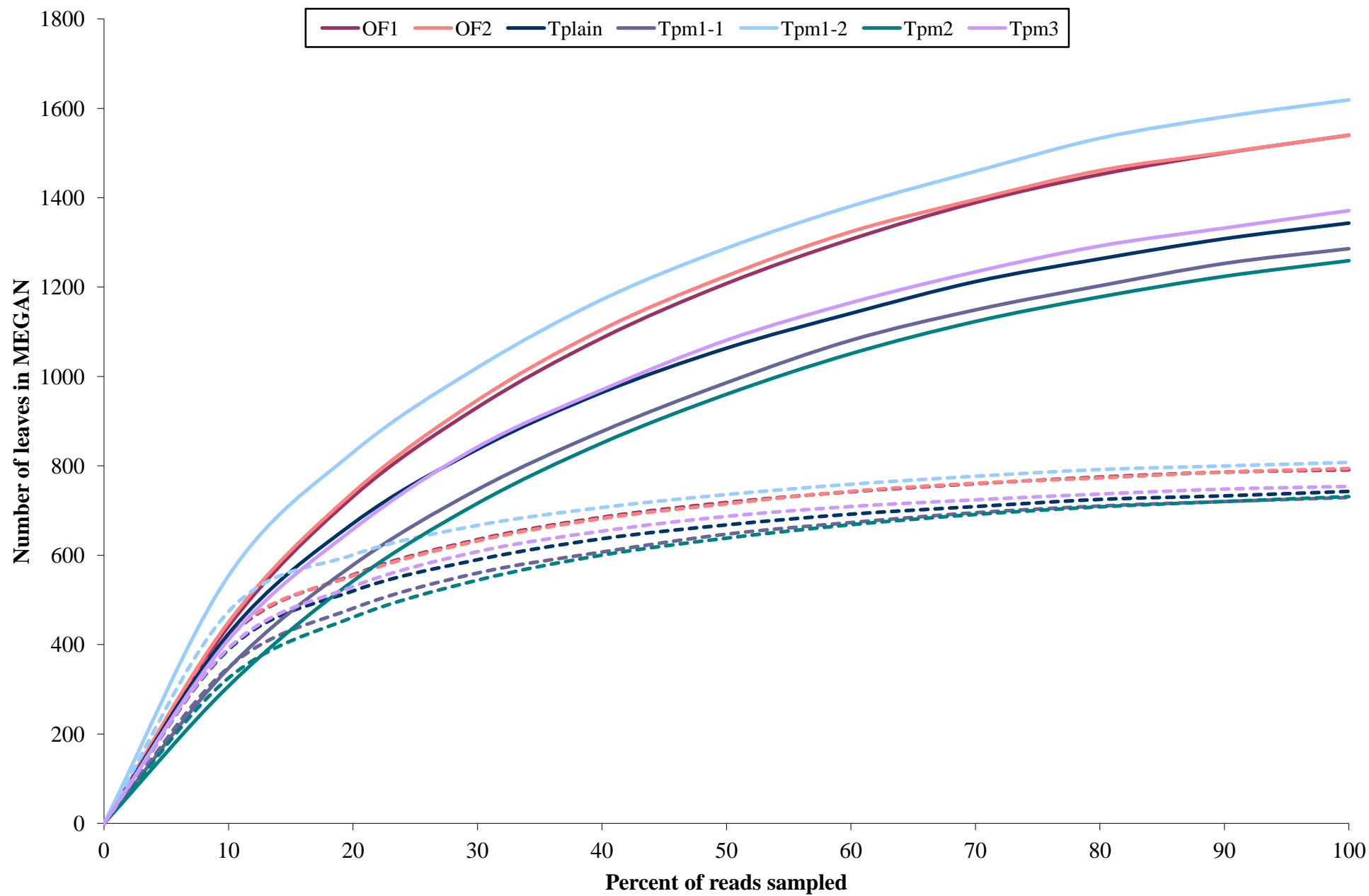

Supplement: Additional file 3 — Figure S2. Rarefaction curves created in MEGAN. Rarefaction analysis was performed at the most resolved and genus level of the NCBI taxonomy in MEGAN for each metagenome. The curves included all taxa (Bacteria, Archaea, Eukaryota, viruses and unclassified sequences). The solid lines represent leaves at the most detailed taxonomic level, while the stippled lines represent leaves at the genus level. [file 1471-2180-12-203-S3.pdf]

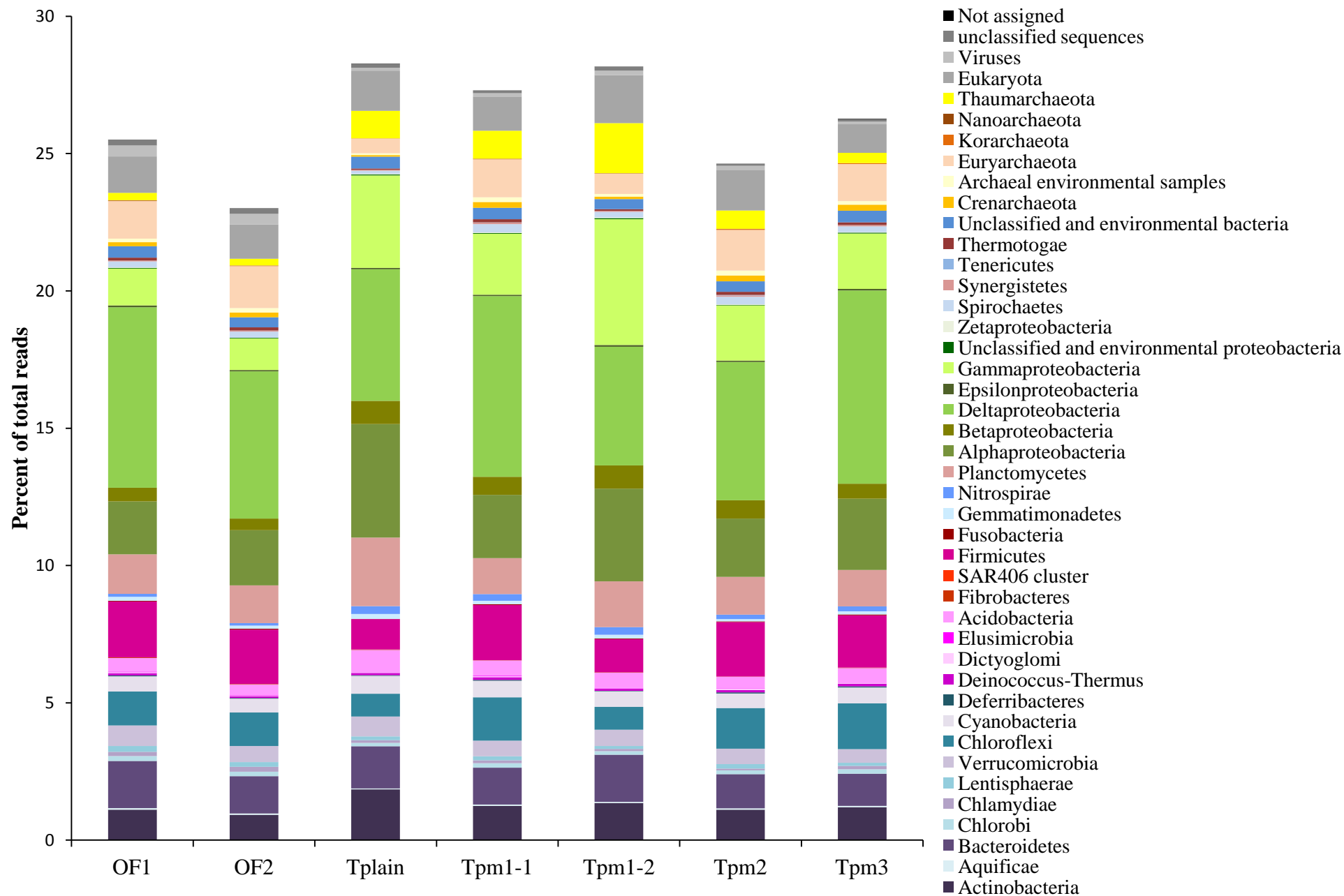

Supplement: Additional file 8 — Figure S4. Taxonomic distribution of prokaryotes based on all reads at the phylum level. The figure shows the taxonomic distribution of prokaryotes in the metagenomes at the phylum level (Proteobacteria are presented at the class level) based on MEGAN analysis (Min Score: 35, Top percent: 10 and Min Support: 5) of all reads after blast against NCBIs non redundant Protein database. [file 1471-2180-12-203-S8.pdf]

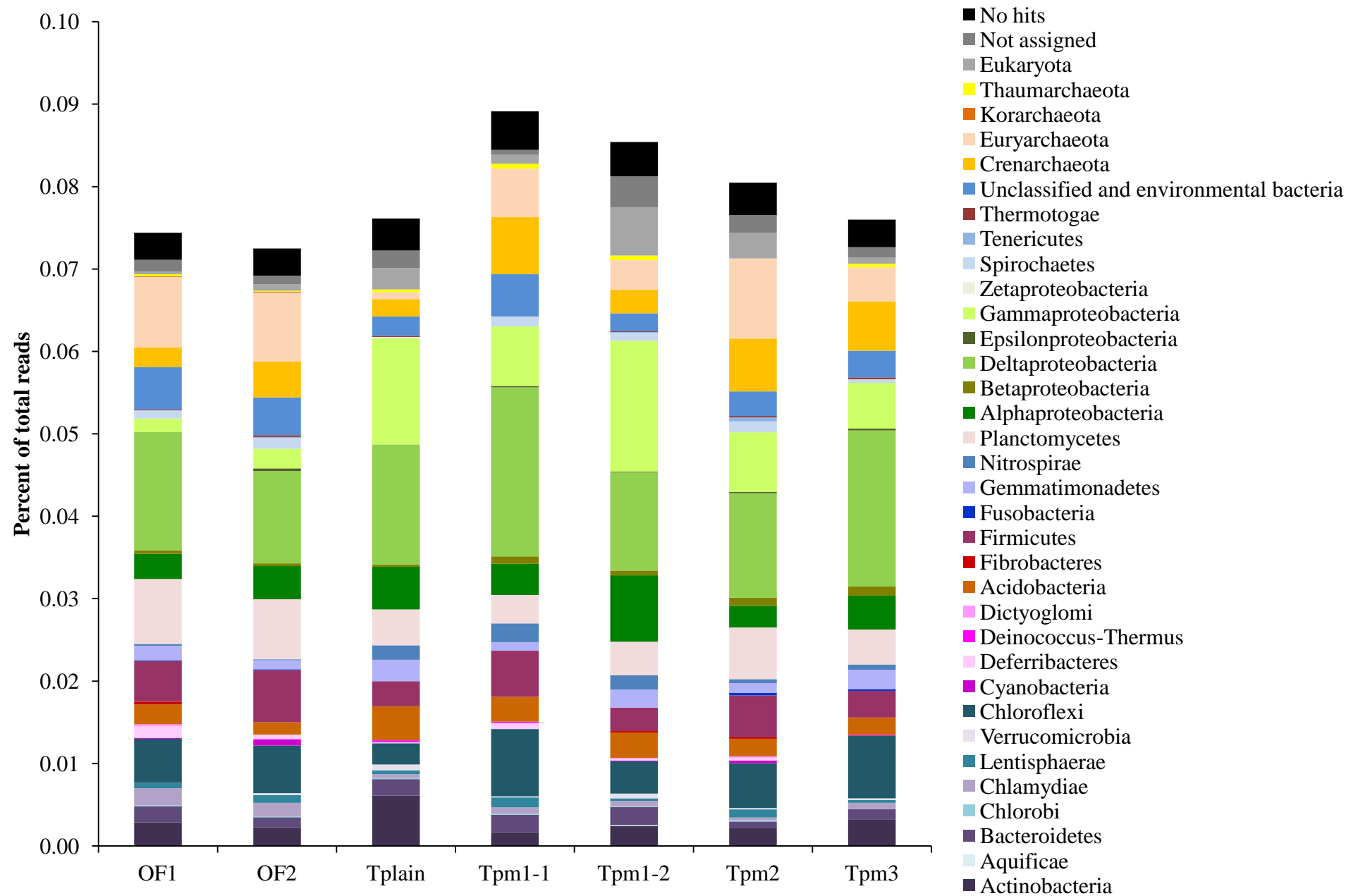

Supplement: Additional file 9 — Figure S5. Taxonomic distribution of prokaryotes based on reads assigned to the 16S rRNA gene at the phylum level. The figure shows the taxonomic distribution of prokaryotes in the metagenomes at the phylum level (Proteobacteria are presented at the class level) based on MEGAN analysis (Min Score: 50, Top percent: 10 and Min Support: 1) of reads assigned to the 16S rRNA gene after blast against the SILVA SSU and LSU databases. [file 1471-2180-12-203-S9.pdf]
