# Supplementary material for: Metagenomic and geochemical characterization of pockmarked sediments overlaying the Troll petroleum reservoir in the North Sea
Source: BMC Microbiol. 2012 Sep 11;12:203. doi: 10.1186/1471-2180-12-203 (PMC3478177; doi:10.1186/1471-2180-12-203)
Supplement: Additional file 2 — Table S1. Sample site description and chemical data. The table shows details on sampling location and chemical data obtained by the Norwegian Geotechnical Institute in the Petrogen project [25]. [file 1471-2180-12-203-S2.docx]

### Table S1: Sample site description and chemical data

The table shows details on sampling location and chemical data obtained by the Norwegian Geotechnical Institute in the Petrogen project [[24](#_ENREF_24)].

| **Parameter** | **unit** | **OF1** | **OF2** | **Tplain** | **Tpm1-1** | **Tpm1-2** | **Tpm2** | **Tpm3** |
| --- | --- | --- | --- | --- | --- | --- | --- | --- |
| **Electric conductivity** | µS/cm | 52500 | 52700 | 53700 | 54100 | 53100 | 52600 | 53100 |
| **NH3-N** | mg/l | 5.35 | 3.45 | 0.03 | 0.558 | 0.542 | 0.934 | 1.27 |
| **NO3+NO2-N** | mg/l | 0.005 | 0.005 | 0.149 | 0.015 | 0.027 | 0.044 | 0.063 |
| **NO3+NO2-N:NH3-N** | ratio | 0.0009 | 0.0014 | 4.9667 | 0.0269 | 0.0498 | 0.0471 | 0.0496 |
| **SO4-S** | mg/l | 862 | 920 | 953 | 946 | 929 | 875 | 930 |
| **Cl** | mg/l | 20400 | 20900 | 21300 | 20200 | 20400 | 19800 | 20000 |
| **TOC** | % | 1.39 | 1.46 | 1.08 | 0.54 | 0.64 | 0.7 | 0.67 |
| **HCO3-C** | mg/l | 459 | 383.5 | 124 | 145 | 124 | 194 | 115 |
| **Ca** | mg/l | 362 | 370 | 400 | 397 | 417 | 389 | 414 |
| **K** | mg/l | 396 | 405 | 478 | 545 | 485 | 497 | 508 |
| **Mg** | mg/l | 1250 | 1260 | 1260 | 1250 | 1270 | 1210 | 1290 |
| **Na** | mg/l | 10800 | 10800 | 10800 | 11000 | 10700 | 10500 | 10900 |
| **S** | mg/l | 881 | 961 | 926 | 951 | 929 | 868 | 934 |
| **Si** | mg/l | 8.03 | 9.01 | 2.91 | 3.66 | 2.58 | 4.12 | 2.93 |
| **Fe** | µg/l | 109 | 112 | 2610 | 738 | 55.7 | 9.11 | 138 |
| **Sr** | µg/l | 7710 | 7840 | 7450 | 7570 | 7600 | 7420 | 7930 |
| **Al** | µg/l | 7.07 | 1.2 | 55.2 | 11.7 | 7.76 | 181 | 27.5 |
| **Ba** | µg/l | 41.9 | 32.4 | 21.3 | 35.5 | 25.9 | 33.7 | 27.6 |
| **Cd** | µg/l | 0.05 | 0.05 | 7.64 | 2.64 | 2.55 | 1.25 | 0.586 |
| **Co** | µg/l | 0.154 | 0.105 | 1.16 | 0.813 | 0.879 | 0.55 | 0.255 |
| **Cr** | µg/l | 0.1 | 0.1 | 1.1 | 0.666 | 0.291 | 0.484 | 0.558 |
| **Cu** | µg/l | 0.5 | 0.5 | 4.5 | 2.12 | 3.97 | 1.56 | 9.81 |
| **Hg** | µg/l | 0.01 | 0.01 | 0.0147 | 0.0052 | 0.0038 | 0.0091 | 0.004 |
| **Mn** | µg/l | 7890 | 5750 | 2200 | 1670 | 1010 | 1070 | 749 |
| **Mo** | µg/l | 81.6 | 71 | 45.4 | 31.3 | 28.1 | 46.9 | 24.6 |
| **Ni** | µg/l | 3.85 | 4.44 | 13.4 | 6.63 | 3.34 | 4.48 | 3.97 |
| **P** | µg/l | 863 | 853 | 383 | 268 | 203 | 230 | 234 |
| **Pb** | µg/l | 0.951 | 1.23 | 9.66 | 6.99 | 11.6 | 10.2 | 5.4 |
| **Zn** | µg/l | 2 | 2 | 18.2 | 18.6 | 26.3 | 18.7 | 16.7 |
| **n-C10** | µg/kg | 0 | 0 | 3.85 | 7.9 | 8.47 | 19 | 16.8 |
| **n-C11** | µg/kg | 0 | 0 | 7.19 | 17.6 | 16.4 | 48.4 | 44.2 |
| **n-C12** | µg/kg | 0 | 0 | 3.29 | 5.78 | 9.02 | 15.7 | 15.9 |
| **i-C13** | µg/kg | 0 | 0 | 1.93 | 2.64 | 2.21 | 4.73 | 5.32 |
| **i-C14** | µg/kg | 0 | 0 | 1.58 | 1.89 | 3.34 | 4.97 | 6.03 |
| **n-C13** | µg/kg | 0 | 0 | 13.4 | 11.3 | 16 | 22.3 | 22.5 |
| **i-C15** | µg/kg | 0 | 0 | 0 | 0 | 8.44 | 13.4 | 11.7 |
| **n-C14** | µg/kg | 0 | 2.49 | 29.4 | 22.6 | 20.4 | 32.5 | 36 |
| **i-C16** | µg/kg | 0 | 2.5 | 7.7 | 6.7 | 9.09 | 14.6 | 14.7 |
| **n-C15** | µg/kg | 0 | 5.16 | 28.8 | 28.3 | 24.4 | 40.2 | 44 |
| **n-C16** | µg/kg | 37.6 | 44.6 | 27 | 30.2 | 31.6 | 47 | 52.7 |
| **i-C18** | µg/kg | 10.5 | 16.5 | 8.32 | 7.32 | 17 | 29.5 | 26.4 |
| **n-C17** | µg/kg | 47.4 | 53.8 | 25.7 | 33.6 | 42 | 55.4 | 56.1 |
| **Pr** | µg/kg | 31.5 | 34 | 23.4 | 28.8 | 39.2 | 55.5 | 45.8 |
| **n-C18** | µg/kg | 35.6 | 40.5 | 28.8 | 33.2 | 41.6 | 49.5 | 45.6 |
| **Ph** | µg/kg | 29.3 | 45.9 | 20.1 | 31 | 35.6 | 42.1 | 27 |
| **n-C19** | µg/kg | 16.9 | 20.2 | 29.6 | 41.9 | 50.2 | 54.6 | 45.5 |
| **n-C20** | µg/kg | 19.2 | 26.3 | 32.9 | 52.1 | 63.4 | 62 | 63.5 |
| **n-C21** | µg/kg | 14.9 | 11.1 | 42.8 | 128 | 84.7 | 106 | 116 |
| **n-C22** | µg/kg | 22.3 | 9.21 | 47.9 | 258 | 98.6 | 137 | 153 |
| **n-C23** | µg/kg | 19.1 | 9 | 78.7 | 488 | 199 | 292 | 318 |
| **n-C24** | µg/kg | 26.2 | 17 | 73.7 | 532 | 130 | 229 | 253 |
| **n-C25** | µg/kg | 13.9 | 4.49 | 98.2 | 619 | 293 | 508 | 463 |
| **n-C26** | µg/kg | 13.5 | 5.85 | 75.2 | 517 | 140 | 302 | 278 |
| **n-C27** | µg/kg | 13 | 5.07 | 113 | 595 | 370 | 614 | 563 |
| **n-C28** | µg/kg | 11.7 | 2.84 | 66.2 | 340 | 108 | 237 | 201 |
| **n-C29** | µg/kg | 14.6 | 2.67 | 151 | 515 | 421 | 644 | 594 |
| **n-C30** | µg/kg | 13.7 | 5.78 | 53 | 187 | 75.3 | 172 | 113 |
| **n-C31** | µg/kg | 20.5 | 2.74 | 183 | 451 | 482 | 695 | 657 |
| **n-C32** | µg/kg | 20.2 | 0 | 0 | 0 | 0 | 0 | 0 |
| **n-C33** | µg/kg | 26.2 | 0 | 0 | 0 | 0 | 0 | 0 |
| **n-C34** | µg/kg | 31.1 | 0 | 0 | 0 | 0 | 0 | 0 |
| **n-C35** | µg/kg | 39.1 | 0 | 0 | 0 | 0 | 0 | 0 |
| **n-C36** | µg/kg | 58.7 | 0 | 0 | 0 | 0 | 0 | 0 |
| **Sum C10-C36** | µg/kg | 587 | 368 | 1276 | 4993 | 2840 | 4547 | 4289 |
