# Supplementary material for: Metagenomic and geochemical characterization of pockmarked sediments overlaying the Troll petroleum reservoir in the North Sea
Source: BMC Microbiol. 2012 Sep 11;12:203. doi: 10.1186/1471-2180-12-203 (PMC3478177; doi:10.1186/1471-2180-12-203)
Supplement: Additional file 4 — Table S2. Score table for the geochemical parameters. The table shows the scores of the geochemical parameters fitted onto the PCA ordination shown in Figure 3. The first two columns gives the direction cosines of the vectors, r2 gives the squared correlation coefficient. The parameters are sorted by increasing p-values. [file 1471-2180-12-203-S4.doc]

### Table S2: Score table for the geochemical parameters

The table shows the scores of the geochemical parameters fitted onto the PCA ordination shown in Figure 3. The first two columns gives the direction cosines of the vectors, r^2^ gives the squared correlation coefficient. The parameters are sorted by increasing p-values.

| **Parameter** | **PC1** | **PC2** | **r2** | **p-value** |
| --- | --- | --- | --- | --- |
| **Hg** | 0.108545 | 0.994092 | 0.8923 | 0.017 |
| **Ca** | -0.83262 | -0.55385 | 0.8833 | 0.018 |
| **Mo** | 0.692661 | 0.721264 | 0.8761 | 0.024 |
| **Zn** | -0.85079 | -0.52551 | 0.7914 | 0.033 |
| **TOC** | 0.494607 | 0.869117 | 0.8235 | 0.052 |
| **Cd** | -0.87045 | 0.492258 | 0.7631 | 0.053 |
| **Si** | 0.88441 | 0.46671 | 0.7766 | 0.053 |
| **Cl** | -0.38529 | 0.922794 | 0.7428 | 0.069 |
| **HCO3-C** | 0.85365 | 0.520847 | 0.7354 | 0.077 |
| **n-C21** | -0.28599 | -0.95823 | 0.7725 | 0.085 |
| **n-C23** | -0.17166 | -0.98516 | 0.7214 | 0.085 |
| **P** | 0.703189 | 0.711003 | 0.7091 | 0.089 |
| **Co** | -0.99406 | 0.108857 | 0.6949 | 0.093 |
| **n-C20** | -0.3958 | -0.91834 | 0.7595 | 0.096 |
| **n-C22** | -0.18288 | -0.98314 | 0.6785 | 0.104 |
| **n-C25** | -0.12374 | -0.99232 | 0.7136 | 0.112 |
| **n-C27** | -0.14277 | -0.98976 | 0.7164 | 0.122 |
| **Mn** | 0.704436 | 0.709767 | 0.643 | 0.132 |
| **K** | -0.56462 | -0.82535 | 0.6487 | 0.145 |
| **Ba** | 0.987976 | -0.15461 | 0.6231 | 0.146 |
| **n-C29** | -0.21715 | -0.97614 | 0.6734 | 0.153 |
| **NH3-N** | 0.906266 | 0.422708 | 0.587 | 0.154 |
| **Ni** | -0.64024 | 0.768173 | 0.5813 | 0.156 |
| **NO3-NO2-N** | -0.80825 | 0.588846 | 0.5622 | 0.159 |
| **n-C26** | -0.08018 | -0.99678 | 0.5993 | 0.164 |
| **n-C24** | -0.10975 | -0.99396 | 0.5709 | 0.169 |
| **n-C28** | -0.0984 | -0.99515 | 0.5899 | 0.175 |
| **n-C31** | -0.27318 | -0.96196 | 0.6158 | 0.178 |
| **n-C19** | -0.42979 | -0.90293 | 0.6182 | 0.182 |
| **n-C17** | 0.898725 | -0.43851 | 0.5833 | 0.185 |
| **Fe** | -0.62607 | 0.779764 | 0.5039 | 0.218 |
| **Pb** | -0.95311 | -0.30262 | 0.551 | 0.228 |
| **n-C16** | 0.884289 | -0.46694 | 0.4932 | 0.254 |
| **n-C30** | -0.09458 | -0.99552 | 0.5046 | 0.271 |
| **SO4-S** | -0.98462 | -0.17472 | 0.4715 | 0.272 |
| **i-C14** | -0.32369 | -0.94616 | 0.4449 | 0.313 |
| **n-C12** | -0.1891 | -0.98196 | 0.4443 | 0.316 |
| **n-C18** | 0.590632 | -0.80694 | 0.4228 | 0.342 |
| **n-C13** | -0.61797 | -0.7862 | 0.431 | 0.347 |
| **Ph** | 0.938532 | -0.34519 | 0.4097 | 0.347 |
| **Conductivity** | -0.95277 | -0.3037 | 0.4021 | 0.352 |
| **n-C15** | -0.57375 | -0.81903 | 0.3984 | 0.355 |
| **n-C10** | -0.08628 | -0.99627 | 0.4188 | 0.355 |
| **Cu** | -0.80826 | -0.58882 | 0.3776 | 0.356 |
| **i-C13** | -0.2548 | -0.96699 | 0.4015 | 0.367 |
| **n-C11** | 0.051459 | -0.99868 | 0.3903 | 0.375 |
| **Cr** | -0.97375 | 0.227612 | 0.3816 | 0.396 |
| **n-C14** | -0.76552 | -0.64341 | 0.3661 | 0.397 |
| **Pr** | 0.532812 | -0.84623 | 0.3706 | 0.418 |
| **i-C16** | -0.4489 | -0.89358 | 0.349 | 0.435 |
| **n-C32** | 0.722239 | 0.691643 | 0.2537 | 0.446 |
| **n-C33** | 0.722239 | 0.691643 | 0.2537 | 0.446 |
| **n-C34** | 0.722239 | 0.691643 | 0.2537 | 0.446 |
| **n-C35** | 0.722239 | 0.691643 | 0.2537 | 0.446 |
| **n-C36** | 0.722239 | 0.691643 | 0.2537 | 0.446 |
| **i-C15** | -0.0675 | -0.99772 | 0.3037 | 0.465 |
| **i-C18** | 0.572485 | -0.81992 | 0.2527 | 0.546 |
| **Mg** | -0.95547 | -0.29509 | 0.2157 | 0.619 |
| **Sr** | 0.81051 | -0.58573 | 0.1579 | 0.698 |
| **S** | -0.81541 | -0.57889 | 0.0956 | 0.831 |
| **Na** | -0.59853 | -0.8011 | 0.0541 | 0.865 |
| **Al** | 0.999946 | 0.010423 | 0.0358 | 0.989 |
