# Supplementary material for: Metagenomic and geochemical characterization of pockmarked sediments overlaying the Troll petroleum reservoir in the North Sea
Source: BMC Microbiol. 2012 Sep 11;12:203. doi: 10.1186/1471-2180-12-203 (PMC3478177; doi:10.1186/1471-2180-12-203)
Supplement: Additional file 5 — Table S3. Metagenomic parameter scores. The table shows metagenomic parameters scores for the first and second principal component in the PCA analysis. [file 1471-2180-12-203-S5.docx]

### Table S3: Metagenomic parameter scores

The table shows metagenomic parameters scores for the first and second principal component in the PCA analysis.

| Parameter | PC1 | Parameter | PC2 |
| --- | --- | --- | --- |
| Proteobacteria | -2.4139 | **Chloroflexi** | -0.1765 |
| Thaumarchaeota | -0.4256 | **Euryarchaeota** | -0.1217 |
| Planctomycetes | -0.2467 | **Thaumarchaeota** | -0.1203 |
| Actinobacteria | -0.2129 | **Firmicutes** | -0.1133 |
| Acidobacteria | -0.0980 | **Proteobacteria** | -0.0776 |
| Clustering-based subsystems | -0.0924 | **Crenarchaeota** | -0.0359 |
| Regulation and Cell signaling | -0.0754 | **Spirochaetes** | -0.0351 |
| Virulence | -0.0730 | **Respiration** | -0.0203 |
| Nitrospirae | -0.0664 | **archaeal environmental samples** | -0.0197 |
| Bacteroidetes | -0.0482 | **Ktedonobacteria** | -0.0121 |
| Amino Acids and Derivatives | -0.0422 | **Thermotogae** | -0.0096 |
| Unclassified | -0.0324 | **Membrane Transport** | -0.0081 |
| Cyanobacteria | -0.0270 | **Protein Metabolism** | -0.0074 |
| Metabolism of Aromatic Compounds | -0.0250 | **Nitrospirae** | -0.0070 |
| Stress Response | -0.0235 | **Synergistetes** | -0.0061 |
| Chordata | -0.0231 | **Thermobaculum** | -0.0055 |
| unclass. Sequences | -0.0223 | **Dictyoglomi** | -0.0050 |
| Nitrogen Metabolism | -0.0202 | **Deinococcus-Thermus** | -0.0044 |
| Gemmatimonadetes | -0.0182 | **Korarchaeota** | -0.0041 |
| Fatty Acids and Lipids | -0.0174 | **Fusobacteria** | -0.0028 |
| Sulfur Metabolism | -0.0157 | **Deferribacteres** | -0.0023 |
| Echinodermata | -0.0112 | **Aquificae** | -0.0020 |
| Potassium metabolism | -0.0110 | **Chlorobi** | -0.0020 |
| Phosphorus Metabolism | -0.0087 | **c. div. WWE1** | -0.0013 |
| Candidatus Poribacteria | -0.0067 | **Oomycetes** | -0.0007 |
| Cnidaria | -0.0050 | **Ciliophora** | -0.0007 |
| Miscellaneous | -0.0048 | **Heterolobosea** | -0.0006 |
| c. div. NC10 | -0.0045 | **DNA Metabolism** | -0.0005 |
| Arthropoda | -0.0044 | **Mollusca** | -0.0004 |
| Nematoda | -0.0037 | **Tenericutes** | -0.0002 |
| Streptophyta | -0.0037 | **Nanoarchaeota** | -0.0002 |
| Bacillariophyta | -0.0036 | **Microsporidia** | -0.0002 |
| Hemichordata | -0.0033 | **Fornicata** | -0.0001 |
| Macromolecular Synthesis | -0.0032 | **Photosynthesis** | -0.0001 |
| bacterial environmental samples | -0.0032 | **Placozoa** | -0.0001 |
| Kinetoplastida | -0.0021 | **c. div. WS3** | -0.0001 |
| Deinococcus-Thermus | -0.0011 | **Basidiomycota** | -0.0001 |
| Secondary Metabolism | -0.0009 | **Bryozoa** | -0.0001 |
| Mollusca | -0.0009 | **Platyhelminthes** | 0.0000 |
| Platyhelminthes | -0.0004 | **Blastocystis** | 0.0000 |
| Chlorophyta | -0.0004 | **Cryptophyta** | 0.0000 |
| Cofactors-Vitamins-Prosthetic groups_Pigments | -0.0003 | **Porifera** | 0.0000 |
| Dinophyceae | -0.0003 | **c. div. WWE3** | 0.0000 |
| Basidiomycota | -0.0002 | **Rotifera** | 0.0001 |
| Placozoa | -0.0002 | **Echinodermata** | 0.0001 |
| Bryozoa | -0.0001 | **Parabasalia** | 0.0002 |
| Microsporidia | -0.0001 | **Annelida** | 0.0002 |
| Fornicata | -0.0001 | **Secondary Metabolism** | 0.0003 |
| Annelida | 0.0000 | **Dinophyceae** | 0.0004 |
| Porifera | 0.0001 | **Amoebozoa** | 0.0004 |
| Rotifera | 0.0001 | **c. div. NC10** | 0.0004 |
| Cryptophyta | 0.0001 | **Perkinsea** | 0.0004 |
| Perkinsea | 0.0002 | **Prophage** | 0.0004 |
| Oomycetes | 0.0002 | **c. div. OP8** | 0.0005 |
| Photosynthesis | 0.0002 | **Phaeophyceae** | 0.0005 |
| Blastocystis | 0.0003 | **Nematoda** | 0.0008 |
| Phaeophyceae | 0.0003 | **Hemichordata** | 0.0008 |
| c. div. OP8 | 0.0004 | **Miscellaneous** | 0.0008 |
| Cell Division and Cell Cycle | 0.0004 | **Bacillariophyta** | 0.0009 |
| c. div. WS3 | 0.0007 | **c. div. TM7** | 0.0009 |
| Prophage | 0.0008 | **Macromolecular Synthesis** | 0.0010 |
| Tenericutes | 0.0012 | **Fatty Acids and Lipids** | 0.0011 |
| c. div. WWE3 | 0.0012 | **Elusimicrobia** | 0.0013 |
| Ciliophora | 0.0014 | **Potassium metabolism** | 0.0017 |
| Choanoflagellida | 0.0015 | **Cnidaria** | 0.0018 |
| Heterolobosea | 0.0016 | **Nucleosides and Nucleotides** | 0.0019 |
| Verrucomicrobia | 0.0019 | **Kinetoplastida** | 0.0020 |
| c. div. TM7 | 0.0023 | **Choanoflagellida** | 0.0020 |
| Parabasalia | 0.0023 | **Fibrobacteres** | 0.0028 |
| Amoebozoa | 0.0026 | **Apicomplexa** | 0.0029 |
| Nanoarchaeota | 0.0033 | **Streptophyta** | 0.0030 |
| Ascomycota | 0.0034 | **Arthropoda** | 0.0031 |
| Cell Wall and Capsule | 0.0035 | **Nitrogen Metabolism** | 0.0034 |
| Deferribacteres | 0.0037 | **Candidatus Poribacteria** | 0.0037 |
| Motility and Chemotaxis | 0.0037 | **Cell Division and Cell Cycle** | 0.0040 |
| Apicomplexa | 0.0042 | **Ascomycota** | 0.0041 |
| Fibrobacteres | 0.0045 | **Chlorophyta** | 0.0044 |
| Thermobaculum | 0.0048 | **unclass. Sequences** | 0.0047 |
| Korarchaeota | 0.0050 | **Carbohydrates** | 0.0051 |
| Elusimicrobia | 0.0059 | **Cofactors-Vitamins-Prosthetic groups_Pigments** | 0.0055 |
| RNA Metabolism | 0.0060 | **Metabolism of Aromatic Compounds** | 0.0059 |
| Fusobacteria | 0.0060 | **Phosphorus Metabolism** | 0.0061 |
| Aquificae | 0.0066 | **RNA Metabolism** | 0.0064 |
| Nucleosides and Nucleotides | 0.0069 | **bacterial environmental samples** | 0.0072 |
| Dictyoglomi | 0.0074 | **Motility and Chemotaxis** | 0.0078 |
| Synergistetes | 0.0075 | **Stress Response** | 0.0086 |
| Ktedonobacteria | 0.0089 | **Chordata** | 0.0100 |
| c. div. WWE1 | 0.0109 | **Cyanobacteria** | 0.0108 |
| Chlorobi | 0.0160 | **Unclassified** | 0.0111 |
| Membrane Transport | 0.0181 | **Amino Acids and Derivatives** | 0.0116 |
| Thermotogae | 0.0186 | **Chlamydiae** | 0.0132 |
| Chlamydiae | 0.0249 | **Gemmatimonadetes** | 0.0180 |
| Lentisphaerae | 0.0249 | **Sulfur Metabolism** | 0.0183 |
| Carbohydrates | 0.0257 | **Lentisphaerae** | 0.0187 |
| Protein Metabolism | 0.0278 | **Cell Wall and Capsule** | 0.0270 |
| archaeal environmental samples | 0.0337 | **Regulation and Cell signaling** | 0.0362 |
| Spirochaetes | 0.0354 | **Viruses** | 0.0463 |
| Respiration | 0.0389 | **Virulence** | 0.0505 |
| DNA Metabolism | 0.0411 | **Acidobacteria** | 0.0517 |
| Crenarchaeota | 0.0417 | **Clustering-based subsystems** | 0.0611 |
| Viruses | 0.0794 | **Verrucomicrobia** | 0.0768 |
| Chloroflexi | 0.1954 | **Bacteroidetes** | 0.1028 |
| Euryarchaeota | 0.3605 | **Actinobacteria** | 0.1117 |
| Firmicutes | 0.3654 | **Planctomycetes** | 0.2862 |
