# Supplementary material for: Metagenomic and geochemical characterization of pockmarked sediments overlaying the Troll petroleum reservoir in the North Sea
Source: BMC Microbiol. 2012 Sep 11;12:203. doi: 10.1186/1471-2180-12-203 (PMC3478177; doi:10.1186/1471-2180-12-203)
Supplement: Additional file 6 — Figure S3. PCA plot showing all measured geochemical parameters. The figure shows the same PCA plot as Figure 3, but displays all the measured geochemical parameters labeled by numbers. [file 1471-2180-12-203-S6.pdf]

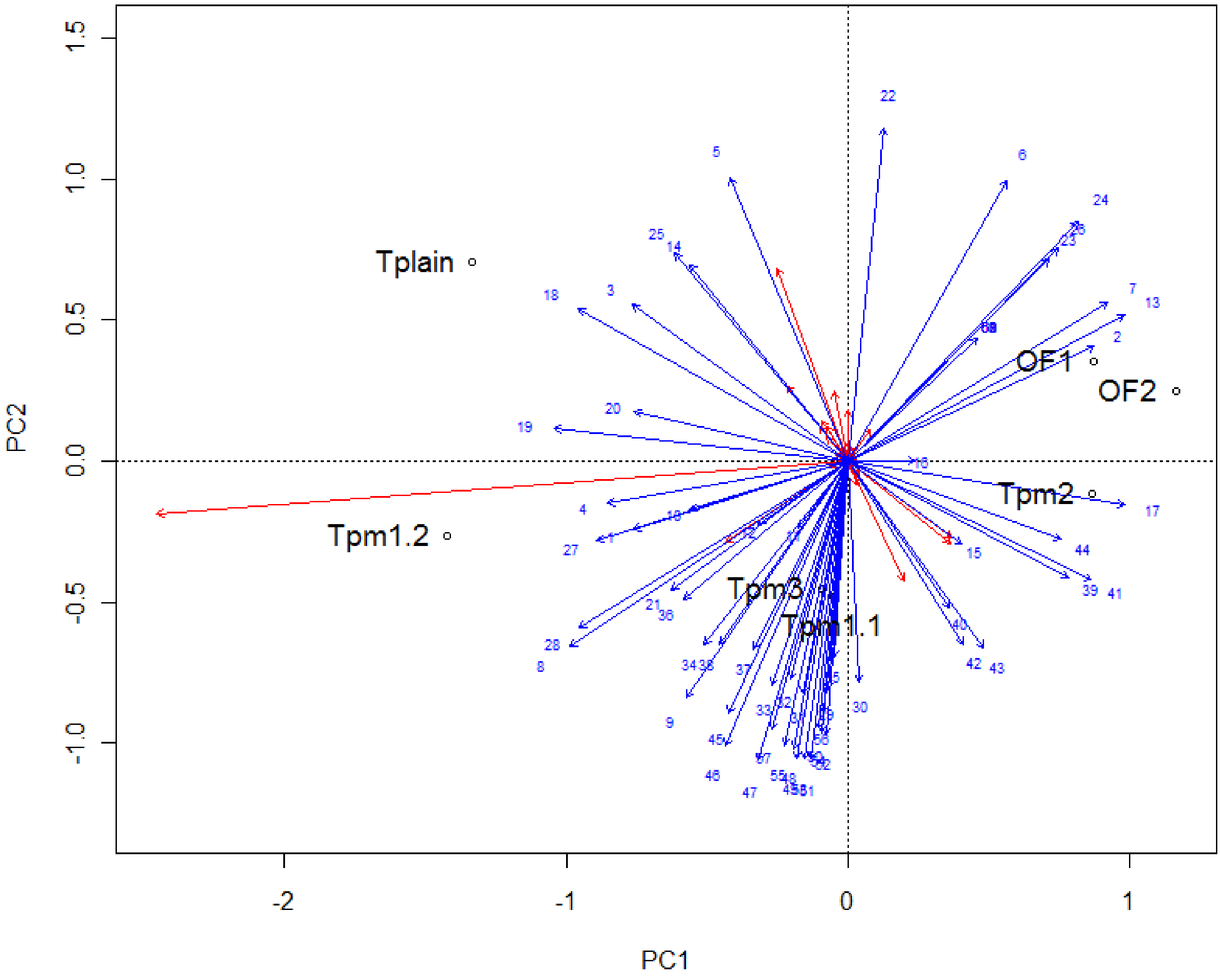

**1**	Conductivity	**17**	Ba	**33**	i-C14	**49**	n-C23
**2**	NH3-N	**18**	Cd	**34**	n-C13	**50**	n-C24
**3**	NO3-NO2-N	**19**	Co	**35**	i-C15	**51**	n-C25
**4**	SO4-S	**20**	Cr	**36**	n-C14	**52**	n-C26
**5**	Cl	**21**	Cu	**37**	i-C16	**53**	n-C27
**6**	TOC	**22**	Hg	**38**	n-C15	**54**	n-C28
**7**	HCO3-C	**23**	Mn	**39**	n-C16	**55**	n-C29
**8**	Ca	**24**	Mo	**40**	i-C18	**56**	n-C30
**9**	K	**25**	Ni	**41**	n-C17	**57**	n-C31
**10**	Mg	**26**	P	**42**	Pr	**58**	n-C32
**11**	Na	**27**	Pb	**43**	n-C18	**59**	n-C33
**12**	S	**28**	Zn	**44**	Ph	**60**	n-C34
**13**	Si	**29**	n-C10	**45**	n-C19	**61**	n-C35
**14**	Fe	**30**	n-C11	**46**	n-C20	**62**	n-C36
**15**	Sr	**31**	n-C12	**47**	n-C21		
**16**	Al	**32**	i-C13	**48**	n-C22		
