# Supplementary material for: Metagenomic and geochemical characterization of pockmarked sediments overlaying the Troll petroleum reservoir in the North Sea
Source: BMC Microbiol. 2012 Sep 11;12:203. doi: 10.1186/1471-2180-12-203 (PMC3478177; doi:10.1186/1471-2180-12-203)
Supplement: Additional file 7 — Table S4. Reads assigned at the domain level in MEGAN. Numbers are given as percent of total reads (numbers based on the reads assigned to the 16S rRNA gene). [file 1471-2180-12-203-S7.docx]

## Table S4: Reads assigned at the domain level in MEGAN

Numbers are given as percent of total reads (numbers based on the reads assigned to the 16S rRNA gene).

|  | **OF1** | **OF2** | **Tplain** | **Tpm1-1** | **Tpm1-2** | **Tpm2** | **Tpm3** |
| --- | --- | --- | --- | --- | --- | --- | --- |
| **Root** | 1.59  (1.6 ∙ 10^-3^) | 1.54  (0.8 ∙ 10^-3^) | 1.59  (1.1 ∙ 10^-3^) | 1.45  (0.9 ∙ 10^-3^) | 1.63  (0.8 ∙ 10^-3^) | 1.49  (0.7 ∙ 10^-3^) | 1.23  (0.3 ∙ 10^-3^) |
| **Cellular organisms** | 8.31  (3.5 ∙ 10^-3^) | 8.63  (2.8 ∙ 10^-3^) | 7.40  (2.1 ∙ 10^-3^) | 9.06  (2.7 ∙ 10^-3^) | 7.25  (2.0 ∙ 10^-3^) | 8.89  (4.4 ∙ 10^-3^) | 8.60  (1.9 ∙ 10^-3^) |
| **Bacteria** | 43.34  (78.5 ∙ 10^-3^) | 41.79  (75.7 ∙ 10^-3^) | 50.29  (84.1 ∙ 10^-3^) | 46.72  (90.5 ∙ 10^-3^) | 46.97  (83.7 ∙ 10^-3^) | 41.46  (75.7 ∙ 10^-3^) | 46.01  (83.4 ∙ 10^-3^) |
| **Archaea** | 2.50  (12.9 ∙ 10^-3^) | 3.14  (15.0 ∙ 10^-3^) | 1.86  (5.4 ∙ 10^-3^) | 3.48  (16.6 ∙ 10^-3^) | 3.08  (10.3 ∙ 10^-3^) | 3.33  (20.6 ∙ 10^-3^) | 2.79  (13.1 ∙ 10^-3^) |
| **Eukaryota** | 1.33  (0.0 ∙ 10^-3^) | 1.37  (0.8 ∙ 10^-3^) | 1.46  (2.6 ∙ 10^-3^) | 1.25  (1.1 ∙ 10^-3^) | 1.75  (5.9 ∙ 10^-3^) | 1.46  (3.0 ∙ 10^-3^) | 1.04  (0.8 ∙ 10^-3^) |
| **Viruses** | 0.40  (0.0 ∙ 10^-3^) | 0.42  (0.0 ∙ 10^-3^) | 0.11  (0.0 ∙ 10^-3^) | 0.12  (0.0 ∙ 10^-3^) | 0.16  (0.0 ∙ 10^-3^) | 0.17  (0.0 ∙ 10^-3^) | 0.10  (0.0 ∙ 10^-3^) |
| **Unclassified sequences** | 0.20  (0.0 ∙ 10^-3^) | 0.21  (0.0 ∙ 10^-3^) | 0.14  (0.0 ∙ 10^-3^) | 0.09  (0.0 ∙ 10^-3^) | 0.14  (0.0 ∙ 10^-3^) | 0.08  (0.0 ∙ 10^-3^) | 0.09  (0.0 ∙ 10^-3^) |
| **Not assigned** | 0.01  (1.4 ∙ 10^-3^) | 0.01  (1.1 ∙ 10^-3^) | 0.01  (2.1 ∙ 10^-3^) | 0.01  (0.6 ∙ 10^-3^) | 0.01  (3.7 ∙ 10^-3^) | 0.00  (2.1 ∙ 10^-3^) | 0.01  (1.2 ∙ 10^-3^) |
| **No hits** | 42.32  (3.3 ∙ 10^-3^) | 42.90  (3.3 ∙ 10^-3^) | 37.15  (3.9 ∙ 10^-3^) | 37.82  (4.7 ∙ 10^-3^) | 39.02  (4.2 ∙ 10^-3^) | 43.12  (4.0 ∙ 10^-3^) | 40.14  (3.3 ∙ 10^-3^) |
