# Supplementary material for: Metagenomic and geochemical characterization of pockmarked sediments overlaying the Troll petroleum reservoir in the North Sea
Source: BMC Microbiol. 2012 Sep 11;12:203. doi: 10.1186/1471-2180-12-203 (PMC3478177; doi:10.1186/1471-2180-12-203)
Supplement: Additional file 10 — Table S5. Significantly over or underrepresented genera in Troll metagenomes compared to both Oslofjord metagenomes. Genera differing significantly in one or more Troll metagenomes compared to both Oslofjord metagenomes after statistical analysis in STAMP. [file 1471-2180-12-203-S10.docx]

### Table S5: Significantly over or underrepresented genera in Troll metagenomes compared to both Oslofjord metagenomes

Genera differing significantly in one or more Troll metagenomes compared to both Oslofjord metagenomes after statistical analysis in STAMP.

| **Underrepresented** | | | **Overrepresented** | | |
| --- | --- | --- | --- | --- | --- |
| **Phylum/Class** | **Genus** | **Metagenomes** | **Phylum/Class** | **Genus** | **Metagenomes** |
| **Actinobacteria** | Eggerthella | Tplain and Tpm1-2 | **Acidobacteria** | environmental samples, Acidobacteria | Tplain |
| **Alphaproteobacteria** | Rickettsia | Tpm2 | **Actinobacteria** | Frankia | Tplain |
| **Alphaproteobacteria** | Gluconacetobacter | Tplain, Tpm1-1, Tpm1-2, Tpm2 and Tpm3 | **Actinobacteria** | Nakamurella | Tplain |
| **Alphaproteobacteria** | Hyphomicrobium | Tpm1-1, Tpm1-2 and Tpm2 | **Actinobacteria** | Sanguibacter | Tplain |
| **Alphaproteobacteria** | alpha proteobacterium HIMB114 | Tpm1-2 and Tpm3 | **Actinobacteria** | Acidimicrobium | Tplain and Tpm1-2 |
| **Aquificae** | Persephonella | Tplain | **Actinobacteria** | Janibacter | Tplain and Tpm1-2 |
| **Aquificae** | Hydrogenobacter | Tplain and Tpm1-2 | **Alphaproteobacteria** | Acidiphilium | Tplain |
| **Bacteroidetes** | Bacteroides | Tpm1-2 | **Alphaproteobacteria** | Aurantimonas | Tplain |
| **Bacteroidetes** | Sphingobacterium | Tpm3 | **Alphaproteobacteria** | Citreicella | Tplain |
| **Bacteroidetes** | Psychroflexus | Tplain, Tpm1-1, Tpm1-2, Tpm2 and Tpm3 | **Alphaproteobacteria** | Octadecabacter | Tplain |
| **Bacteroidetes** | Blattabacterium | Tplain, Tpm1-2, Tpm2 and Tpm3 | **Alphaproteobacteria** | Roseobacter | Tplain |
| **Bacteroidetes** | Alistipes | Tpm1-2 and Tpm3 | **Alphaproteobacteria** | Sphingobium | Tplain |
| **Betaproteobacteria** | Delftia | Tpm1-1 | **Alphaproteobacteria** | unclassified Rhodobacteraceae | Tplain |
| **Betaproteobacteria** | Kingella | Tpm1-2 | **Alphaproteobacteria** | unclassified Rhodobacterales | Tplain |
| **Betaproteobacteria** | Comamonas | Tpm1-1 and Tpm3 | **Alphaproteobacteria** | Hyphomonas | Tpm1-2 |
| **Chlamydiae** | Candidatus Protochlamydia | Tpm2 | **Alphaproteobacteria** | Sagittula | Tpm1-2 |
| **Chlamydiae** | Parachlamydia | Tpm2 | **Alphaproteobacteria** | Azospirillum | Tplain and Tpm1-2 |
| **Chloroflexi** | Dehalococcoides | Tplain and Tpm1-2 | **Alphaproteobacteria** | environmental samples, Rhodospirillales | Tplain and Tpm1-2 |
| **Chloroflexi** | Dehalogenimonas | Tplain and Tpm1-2 | **Alphaproteobacteria** | Erythrobacter | Tplain and Tpm1-2 |
| **Crenarchaeota** | Desulfurococcus | Tplain | **Alphaproteobacteria** | Jannaschia | Tplain and Tpm1-2 |
| **Crenarchaeota** | Ignisphaera | Tplain | **Alphaproteobacteria** | Oceanicaulis | Tplain and Tpm1-2 |
| **Crenarchaeota** | Staphylothermus | Tplain | **Alphaproteobacteria** | Parvibaculum | Tplain and Tpm1-2 |
| **Crenarchaeota** | Sulfolobus | Tplain | **Alphaproteobacteria** | Parvularcula | Tplain and Tpm1-2 |
| **Crenarchaeota** | Acidilobus | Tplain and Tpm1-2 | **Alphaproteobacteria** | Phenylobacterium | Tplain and Tpm1-2 |
| **Crenarchaeota** | Caldivirga | Tplain and Tpm1-2 | **Alphaproteobacteria** | Roseovarius | Tplain and Tpm1-2 |
| **Crenarchaeota** | Hyperthermus | Tplain and Tpm1-2 | **Alphaproteobacteria** | Sphingomonas | Tplain and Tpm1-2 |
| **Crenarchaeota** | Thermofilum | Tplain and Tpm1-2 | **Alphaproteobacteria** | Sulfitobacter | Tplain and Tpm1-2 |
| **Crenarchaeota** | Thermosphaera | Tplain and Tpm1-2 | **Alphaproteobacteria** | Thalassobium | Tplain and Tpm1-2 |
| **Cyanobacteria** | Thermosynechococcus | Tpm1-2 | **Alphaproteobacteria** | unclassified Alphaproteobacteria (miscellaneous) | Tplain and Tpm1-2 |
| **Deltaproteobactereia** | Desulfatibacillum | Tpm1-2 | **Alphaproteobacteria** | Maricaulis | Tplain, Tpm1-1 and Tpm1-2 |
| **Deltaproteobactereia** | Desulfobacterium | Tpm1-2 | **Alphaproteobacteria** | Rhodospirillum | Tplain, Tpm1-1 and Tpm1-2 |
| **Deltaproteobactereia** | Desulfococcus | Tpm1-2 | **Alphaproteobacteria** | Ketogulonicigenium | Tplain, Tpm1-2 and Tpm2 |
| **Deltaproteobactereia** | Syntrophobacter | Tpm1-2 | **Bacteroidetes** | Polaribacter | Tpm1-2 |
| **Deltaproteobactereia** | unclassified Deltaproteobacteria (miscellaneous) | Tpm1-2 | **Betaproteobacteria** | Methylibium | Tplain |
| **Deltaproteobactereia** | Lawsonia | Tplain and Tpm1-2 | **Betaproteobacteria** | Polaromonas | Tplain |
| **Elusimicrobia** | Elusimicrobium | Tplain and Tpm1-2 | **Betaproteobacteria** | Hahella | Tpm1-2 |
| **Elusimicrobia** | environmental samples, Elusimicrobia | Tplain, Tpm1-2 and Tpm3 | **Betaproteobacteria** | Leptothrix | Tpm1-2 |
| **Epsilonproteobacteria** | Nitratiruptor | Tplain | **Betaproteobacteria** | Methylotenera | Tpm1-2 |
| **Euryarchaeota** | Archaeoglobus | Tplain | **Betaproteobacteria** | Sideroxydans | Tpm1-2 |
| **Euryarchaeota** | Candidatus Methanoregula | Tplain | **Betaproteobacteria** | Limnobacter | Tplain and Tpm1-2 |
| **Euryarchaeota** | environmental samples, Euryarchaeota | Tplain | **Betaproteobacteria** | Nitrosomonas | Tplain and Tpm1-2 |
| **Euryarchaeota** | Methanococcoides | Tplain | **Betaproteobacteria** | Nitrosospira | Tplain and Tpm1-2 |
| **Euryarchaeota** | Methanococcus | Tplain | **Betaproteobacteria** | Thauera | Tplain and Tpm1-2 |
| **Euryarchaeota** | Methanocorpusculum | Tplain | **Betaproteobacteria** | Thiobacillus | Tplain and Tpm1-2 |
| **Euryarchaeota** | Methanohalobium | Tplain | **Betaproteobacteria** | Cupriavidus | Tplain, Tpm1-1 and Tpm1-2 |
| **Euryarchaeota** | Methanothermobacter | Tplain | **Betaproteobacteria** | Ralstonia | Tpm1-1 and Tpm2 |
| **Euryarchaeota** | Methanothermococcus | Tplain | **Cyanobacteria** | Cyanobium | Tplain |
| **Euryarchaeota** | Pyrococcus | Tplain | **Deltaproteobactereia** | Plesiocystis | Tpm1-2 |
| **Euryarchaeota** | Thermococcus | Tplain | **Deltaproteobactereia** | environmental samples, Desulfobacterales | Tplain and Tpm1-2 |
| **Euryarchaeota** | Thermoplasma | Tplain | **Deltaproteobactereia** | Haliangium | Tplain and Tpm1-2 |
| **Euryarchaeota** | Methanocella | Tpm1-2 | **Gammaproteobacteria** | Methylococcus | Tplain |
| **Euryarchaeota** | Aciduliprofundum | Tplain and Tpm1-2 | **Gammaproteobacteria** | Alcanivorax | Tpm1-2 |
| **Euryarchaeota** | Methanocaldococcus | Tplain and Tpm1-2 | **Gammaproteobacteria** | Allochromatium | Tpm1-2 |
| **Euryarchaeota** | Methanoculleus | Tplain and Tpm1-2 | **Gammaproteobacteria** | Azotobacter | Tpm1-2 |
| **Euryarchaeota** | Methanopyrus | Tplain and Tpm1-2 | **Gammaproteobacteria** | Cellvibrio | Tpm1-2 |
| **Euryarchaeota** | Methanosaeta | Tplain and Tpm1-2 | **Gammaproteobacteria** | Endoriftia | Tpm1-2 |
| **Euryarchaeota** | Candidatus Micrarchaeum | Tplain, Tpm1-1 and Tpm1-2 | **Gammaproteobacteria** | Grimontia | Tpm1-2 |
| **Euryarchaeota** | Candidatus Parvarchaeum | Tplain, Tpm1-2 and Tpm3 | **Gammaproteobacteria** | Halothiobacillus | Tpm1-2 |
| **Fibrobacteres** | Fibrobacter | Tplain, Tpm1-2 and Tpm3 | **Gammaproteobacteria** | Marinobacter | Tpm1-2 |
| **Firmicutes** | Acetivibrio | Tplain | **Gammaproteobacteria** | Marinomonas | Tpm1-2 |
| **Firmicutes** | Anaerococcus | Tplain | **Gammaproteobacteria** | Oceanobacter | Tpm1-2 |
| **Firmicutes** | Butyrivibrio | Tplain | **Gammaproteobacteria** | Pseudoalteromonas | Tpm1-2 |
| **Firmicutes** | Clostridium | Tplain | **Gammaproteobacteria** | Saccharophagus | Tpm1-2 |
| **Firmicutes** | Coprothermobacter | Tplain | **Gammaproteobacteria** | Teredinibacter | Tpm1-2 |
| **Firmicutes** | Enterococcus | Tplain | **Gammaproteobacteria** | Xanthomonas | Tpm1-2 |
| **Firmicutes** | Halothermothrix | Tplain | **Gammaproteobacteria** | Rickettsiella | Tpm3 |
| **Firmicutes** | Listeria | Tplain | **Gammaproteobacteria** | Thioalkalivibrio | Tplain and Tpm1-1 |
| **Firmicutes** | Lysinibacillus | Tplain | **Gammaproteobacteria** | Colwellia | Tplain and Tpm1-2 |
| **Firmicutes** | Peptoniphilus | Tplain | **Gammaproteobacteria** | Congregibacter | Tplain and Tpm1-2 |
| **Firmicutes** | Syntrophothermus | Tplain | **Gammaproteobacteria** | environmental samples, Gammaproteobacteria | Tplain and Tpm1-2 |
| **Firmicutes** | Thermoanaerobacter | Tplain | **Gammaproteobacteria** | Kangiella | Tplain and Tpm1-2 |
| **Firmicutes** | Turicibacter | Tplain | **Gammaproteobacteria** | Nitrococcus | Tplain and Tpm1-2 |
| **Firmicutes** | Anaerostipes | Tpm1-2 | **Gammaproteobacteria** | Nitrosococcus | Tplain and Tpm1-2 |
| **Firmicutes** | Coprococcus | Tpm1-2 | **Gammaproteobacteria** | Oceanospirillum | Tplain and Tpm1-2 |
| **Firmicutes** | Holdemania | Tpm1-2 | **Gammaproteobacteria** | SAR92 clade | Tplain and Tpm1-2 |
| **Firmicutes** | Roseburia | Tpm1-2 | **Gammaproteobacteria** | unclassified Gammaproteobacteria (miscellaneous) | Tplain and Tpm1-2 |
| **Firmicutes** | Thermosinus | Tpm1-2 | **Gammaproteobacteria** | Alkalilimnicola | Tplain, Tpm1-1 and Tpm1-2 |
|  |  |  |  |  |  |
| **Firmicutes** | unclassified Clostridiales (miscellaneous) | Tpm1-2 | **Gammaproteobacteria** | BD1-7 clade | Tplain, Tpm1-1 and Tpm1-2 |
| **Firmicutes** | Anaerofustis | Tplain and Tpm1-2 | **Gammaproteobacteria** | marine gamma proteobacterium HTCC2080 | Tplain, Tpm1-1 and Tpm1-2 |
| **Firmicutes** | Blautia | Tplain and Tpm1-2 | **Gammaproteobacteria** | Nitrospira | Tplain, Tpm1-1 and Tpm1-2 |
| **Firmicutes** | Caldicellulosiruptor | Tplain and Tpm1-2 | **Gammaproteobacteria** | marine gamma proteobacterium HTCC2148 | Tplain, Tpm1-1, Tpm1-2 and Tpm2 |
| **Firmicutes** | Epulopiscium | Tplain and Tpm1-2 | **Gammaproteobacteria** | Methylophaga | Tplain, Tpm1-1, Tpm1-2 and Tpm3 |
| **Firmicutes** | Ethanoligenens | Tplain and Tpm1-2 | **Gammaproteobacteria** | Thiomicrospira | Tpm1-1 and Tpm1-2 |
| **Firmicutes** | Selenomonas | Tplain and Tpm1-2 | **Gammaproteobacteria** | unclassified Oceanospirillales | Tpm1-1 and Tpm1-2 |
| **Firmicutes** | unclassified Erysipelotrichaceae | Tplain and Tpm1-2 | **Nitrospirae** | Leptospira | Tpm1-2 |
| **Firmicutes** | Veillonella | Tplain and Tpm1-2 | **Nitrospirae** | Thermodesulfovibrio | Tpm1-1, Tpm1-2 and Tpm3 |
| **Fusobacteria** | Fusobacterium | Tplain | **Spirochaetes** | Cenarchaeum | Tplain, Tpm1-1 and Tpm1-2 |
| **Fusobacteria** | Sebaldella | Tplain | **Thaumarchaeota** | environmental samples, marine archaeal group 1 | Tplain and Tpm1-2 |
| **Gammaproteobacteria** | Salmonella | Tplain | **Thaumarchaeota** | Nitrosopumilus | Tplain, Tpm1-1, Tpm1-2 and Tpm2 |
| **Gammaproteobacteria** | Halomonas | Tpm1-1 |  |  |  |
| **Lentisphaerae** | Victivallis | Tpm1-2 |  |  |  |
| **Nanoarchaeota** | Nanoarchaeum | Tplain and Tpm3 |  |  |  |
| **SAR406 cluster** | SAR406 cluster | Tplain and Tpm1-2 |  |  |  |
| **Spirochaetes** | Borrelia | Tplain |  |  |  |
| **Spirochaetes** | Brachyspira | Tplain |  |  |  |
| **Spirochaetes** | Spirochaeta | Tplain |  |  |  |
| **Synergistes** | Aminobacterium | Tplain |  |  |  |
| **Synergistes** | Pyramidobacter | Tplain |  |  |  |
| **Tenericutes** | Mycoplasma | Tplain |  |  |  |
| **Thermotogae** | Kosmotoga | Tplain |  |  |  |
| **Thermotogae** | Petrotoga | Tplain |  |  |  |
| **Thermotogae** | Thermosipho | Tplain |  |  |  |
| **unclassified Bacteria** | candidate division OP8 | Tpm1-2 |  |  |  |
| **unclassified Bacteria** | candidate division WWE3 | Tplain and Tpm1-2 |  |  |  |
| **unclassified Bacteria** | Candidatus Cloacamonas | Tplain and Tpm1-2 |  |  |  |
| **Verrucomicrobia** | environmental samples, Verrucomicrobiales | Tplain |  |  |  |
|  | environmental samples, Archaea | Tplain |  |  |  |
