# Supplementary material for: Metagenomic and geochemical characterization of pockmarked sediments overlaying the Troll petroleum reservoir in the North Sea
Source: BMC Microbiol. 2012 Sep 11;12:203. doi: 10.1186/1471-2180-12-203 (PMC3478177; doi:10.1186/1471-2180-12-203)
Supplement: Additional file 11 — Table S6. Abundant bacterial and archaeal taxa at the genus level. Taxa with ≥ 0.1% of the reads in one or more metagenomes are presented. Numbers are given as percent of total reads. [file 1471-2180-12-203-S11.docx]

### Table S6: Abundant bacterial and archaeal taxa at the genus level

Taxa with ≥ 0.1% of the reads in one or more metagenomes are presented.

Numbers are given as percent of total reads.

| **Domain** | **Phylum/Class** | **Genus** | **Tplain** | **Tpm1-1** | **Tpm1-2** | **Tpm2** | **Tpm3** | **OF1** | **OF2** |
| --- | --- | --- | --- | --- | --- | --- | --- | --- | --- |
| **Bacteria** | Acidobacteria | Candidatus Koribacter | 0.12 | 0.09 | 0.08 | 0.07 | 0.09 | 0.08 | 0.07 |
| **Bacteria** | Acidobacteria | Candidatus Solibacter | 0.40 | 0.27 | 0.28 | 0.20 | 0.25 | 0.25 | 0.21 |
| **Bacteria** | Actinobacteria | Frankia | 0.10 | 0.06 | 0.08 | 0.05 | 0.05 | 0.05 | 0.04 |
| **Bacteria** | Actinobacteria | Mycobacterium | 0.12 | 0.10 | 0.10 | 0.12 | 0.08 | 0.09 | 0.09 |
| **Bacteria** | Actinobacteria | Streptomyces | 0.29 | 0.23 | 0.25 | 0.23 | 0.20 | 0.22 | 0.20 |
| **Bacteria** | Alphaproteobacteria | Hyphomicrobium | 0.23 | 0.06 | 0.06 | 0.07 | 0.21 | 0.16 | 0.22 |
| **Bacteria** | Alphaproteobacteria | unclassified Alphaproteobacteria (miscellaneous) | 0.13 | 0.08 | 0.12 | 0.06 | 0.06 | 0.05 | 0.05 |
| **Bacteria** | Bacteria | environmental samples | 0.20 | 0.18 | 0.17 | 0.15 | 0.18 | 0.19 | 0.17 |
| **Bacteria** | Bacteroidetes | Bacteroides | 0.09 | 0.12 | 0.08 | 0.12 | 0.11 | 0.19 | 0.17 |
| **Bacteria** | Bacteroidetes | Rhodothermus | 0.11 | 0.08 | 0.07 | 0.06 | 0.07 | 0.08 | 0.06 |
| **Bacteria** | Betaproteobacteria | Burkholderia | 0.16 | 0.12 | 0.14 | 0.11 | 0.10 | 0.11 | 0.11 |
| **Bacteria** | Chloroflexi | Dehalococcoides | 0.03 | 0.19 | 0.07 | 0.19 | 0.16 | 0.16 | 0.19 |
| **Bacteria** | Chloroflexi | Ktedonobacter | 0.11 | 0.15 | 0.09 | 0.13 | 0.16 | 0.12 | 0.12 |
| **Bacteria** | Chloroflexi | Roseiflexus | 0.12 | 0.17 | 0.10 | 0.18 | 0.20 | 0.14 | 0.13 |
| **Bacteria** | Chloroflexi | Sphaerobacter | 0.07 | 0.09 | 0.05 | 0.07 | 0.10 | 0.06 | 0.07 |
| **Bacteria** | Deltaproteobacteria | Anaeromyxobacter | 0.19 | 0.14 | 0.16 | 0.12 | 0.14 | 0.12 | 0.11 |
| **Bacteria** | Deltaproteobacteria | Desulfatibacillum | 0.16 | 0.27 | 0.14 | 0.23 | 0.30 | 0.33 | 0.30 |
| **Bacteria** | Deltaproteobacteria | Desulfobacterium | 0.35 | 0.70 | 0.31 | 0.54 | 0.79 | 0.75 | 0.66 |
| **Bacteria** | Deltaproteobacteria | Desulfococcus | 0.12 | 0.20 | 0.10 | 0.15 | 0.22 | 0.23 | 0.22 |
| **Bacteria** | Deltaproteobacteria | Desulfovibrio | 0.13 | 0.16 | 0.11 | 0.13 | 0.18 | 0.19 | 0.17 |
| **Bacteria** | Deltaproteobacteria | Geobacter | 0.31 | 0.43 | 0.34 | 0.30 | 0.40 | 0.35 | 0.32 |
| **Bacteria** | Deltaproteobacteria | Haliangium | 0.27 | 0.21 | 0.27 | 0.15 | 0.15 | 0.11 | 0.09 |
| **Bacteria** | Deltaproteobacteria | Plesiocystis | 0.14 | 0.10 | 0.19 | 0.12 | 0.07 | 0.08 | 0.06 |
| **Bacteria** | Deltaproteobacteria | Sorangium | 0.23 | 0.21 | 0.25 | 0.23 | 0.13 | 0.12 | 0.09 |
| **Bacteria** | Deltaproteobacteria | Syntrophobacter | 0.24 | 0.28 | 0.12 | 0.17 | 0.41 | 0.35 | 0.31 |
| **Bacteria** | Deltaproteobacteria | Syntrophus | 0.06 | 0.09 | 0.05 | 0.07 | 0.10 | 0.09 | 0.09 |
| **Bacteria** | Deltaproteobacteria | unclassified Deltaproteobacteria (miscellaneous) | 0.40 | 0.77 | 0.36 | 0.61 | 0.78 | 0.79 | 0.79 |
| **Bacteria** | Firmicutes | Bacillus | 0.08 | 0.12 | 0.09 | 0.13 | 0.13 | 0.14 | 0.14 |
| **Bacteria** | Firmicutes | Clostridium | 0.10 | 0.19 | 0.12 | 0.19 | 0.20 | 0.21 | 0.23 |
| **Bacteria** | Gammaproteobacteria | BD1-7 clade | 0.07 | 0.03 | 0.10 | 0.03 | 0.02 | 0.01 | 0.02 |
| **Bacteria** | Gammaproteobacteria | marine gamma proteobacterium HTCC2148 | 0.15 | 0.07 | 0.23 | 0.07 | 0.05 | 0.03 | 0.02 |
| **Bacteria** | Gammaproteobacteria | Nitrosococcus | 0.13 | 0.09 | 0.13 | 0.07 | 0.08 | 0.06 | 0.06 |
| **Bacteria** | Gammaproteobacteria | Shewanella | 0.09 | 0.07 | 0.11 | 0.06 | 0.07 | 0.06 | 0.05 |
| **Bacteria** | Gammaproteobacteria | unclassified Gammaproteobacteria (miscellaneous) | 0.14 | 0.07 | 0.14 | 0.06 | 0.07 | 0.05 | 0.04 |
| **Bacteria** | Gemmatimonadetes | Gemmatimonas | 0.18 | 0.13 | 0.14 | 0.08 | 0.11 | 0.14 | 0.13 |
| **Bacteria** | Lentisphaerae | Lentisphaera | 0.10 | 0.08 | 0.08 | 0.09 | 0.07 | 0.12 | 0.11 |
| **Bacteria** | Nitrospirae | Nitrospira | 0.24 | 0.13 | 0.18 | 0.10 | 0.09 | 0.06 | 0.05 |
| **Bacteria** | Planctomycetes | Blastopirellula | 0.20 | 0.09 | 0.13 | 0.10 | 0.11 | 0.10 | 0.12 |
| **Bacteria** | Planctomycetes | Candidatus Kuenenia | 0.39 | 0.31 | 0.22 | 0.24 | 0.19 | 0.23 | 0.22 |
| **Bacteria** | Planctomycetes | Gemmata | 0.12 | 0.09 | 0.10 | 0.09 | 0.08 | 0.10 | 0.10 |
| **Bacteria** | Planctomycetes | Pirellula | 0.22 | 0.09 | 0.14 | 0.12 | 0.12 | 0.11 | 0.13 |
| **Bacteria** | Planctomycetes | Planctomyces | 0.50 | 0.25 | 0.35 | 0.26 | 0.26 | 0.30 | 0.29 |
| **Bacteria** | Planctomycetes | Rhodopirellula | 0.17 | 0.08 | 0.12 | 0.09 | 0.08 | 0.09 | 0.10 |
| **Bacteria** | Spirochaetes | Spirochaeta | 0.05 | 0.18 | 0.08 | 0.17 | 0.12 | 0.12 | 0.13 |
| **Bacteria** | Verrucomicrobia | Verrucomicrobia subdivision 3 | 0.15 | 0.13 | 0.12 | 0.12 | 0.11 | 0.17 | 0.15 |
| **Archaea** |  | environmental samples, Archaea | 0.06 | 0.17 | 0.10 | 0.18 | 0.13 | 0.13 | 0.18 |
| **Archaea** | Euryarchaeota | Methanosarcina | 0.05 | 0.10 | 0.06 | 0.09 | 0.09 | 0.10 | 0.11 |
| **Archaea** | Thaumarchaeota | Nitrosopumilus | 0.50 | 0.58 | 1.02 | 0.38 | 0.18 | 0.13 | 0.11 |
