# Supplementary material for: Metagenomic and geochemical characterization of pockmarked sediments overlaying the Troll petroleum reservoir in the North Sea
Source: BMC Microbiol. 2012 Sep 11;12:203. doi: 10.1186/1471-2180-12-203 (PMC3478177; doi:10.1186/1471-2180-12-203)
Supplement: Additional file 12 — Table S7. Relative proportion of reads assigned to SEED subsystems (level I). Abundances are presented as percent of total reads. Subsystems where a Troll metagenome showed significant differences compared to both Oslofjord metagenomes in the STAMP analysis are marked with an asterisk. [file 1471-2180-12-203-S12.docx]

### Table S7: Relative proportion of reads assigned to SEED subsystems (level I)

Abundances are presented as percent of total reads. Subsystems where a Troll metagenome showed significant differences compared to both Oslofjord metagenomes in the STAMP analysis are marked with an asterisk.

| **SEED level I** | **OF1** | **OF2** | **Tplain** | **Tpm1-1** | **Tpm1-2** | **Tpm2** | **Tpm3** |
| --- | --- | --- | --- | --- | --- | --- | --- |
| **Clustering-based subsystems** | 1.907 | 1.905 | 2.109 | 1.969 | 2.013 | 1.659 | 1.769 |
| **Carbohydrates** | 1.756 | 1.740 | 1.697 | 1.894 | 1.556 | 1.557 | 1.645 |
| **Amino Acids and Derivatives** | 1.064 | 1.087 | 1.174 | 1.178 | 1.108 | 0.951 | 1.059 |
| **Protein Metabolism** | 1.013 | 1.053 | 0.909 | 1.076 | 0.948 | 0.905 | 0.919 |
| **Virulence** | 0.905 | 0.877 | 1.079 | 0.929 | 0.962 | 0.726 | 0.828 |
| **Respiration** | 0.839 | 0.850 | 0.732 | 0.921 | 0.694 | 0.730 | 0.834 |
| **Cofactors. Vitamins. Prosthetic Groups. Pigments** | 0.710 | 0.744 | 0.720 | 0.773 | 0.687 | 0.639 | 0.674 |
| **Unclassified** | 0.637 | 0.618 | 0.712 | 0.703 | 0.661 | 0.566 | 0.606 |
| **DNA Metabolism** | 0.616 | 0.651 | 0.504 | 0.611 | 0.525 | 0.559 | 0.536 |
| **Cell Wall and Capsule** | 0.595 | 0.591 | 0.567 | 0.545 | 0.540 | 0.480 | 0.492 |
| **RNA Metabolism** | 0.474 | 0.504 | 0.452 | 0.491 | 0.458 | 0.417 | 0.421 |
| **Nucleosides and Nucleotides** | 0.426 | 0.432 | 0.399 | 0.442 | 0.390 | 0.368 | 0.394 |
| **Membrane Transport** | 0.409 | 0.418 | 0.372 | 0.460 | 0.343 | 0.376 | 0.401 |
| **Regulation and Cell signaling** | 0.395 | 0.386 | 0.618 | 0.461 | 0.493 | 0.325 | 0.399 |
| **Motility and Chemotaxis** | 0.388 | 0.371 | 0.347 | 0.381 | 0.349 | 0.306 | 0.315 |
| **Stress Response** | 0.315 | 0.299 | 0.364 | 0.350 | 0.330 | 0.252 | 0.298 |
| **Cell Division and Cell Cycle** | 0.244 | 0.238 | 0.226 | 0.242 | 0.226 | 0.195 | 0.212 |
| **Sulfur Metabolism** | 0.232 | 0.208 | 0.270 | 0.217 | 0.217 | 0.171 | 0.201 |
| **Phosphorus Metabolism** | 0.158 | 0.170 | 0.183 | 0.173 | 0.171 | 0.133 | 0.147 |
| **Metabolism of Aromatic Compounds** | 0.145 | 0.145 | 0.210 | 0.185 | 0.194 | 0.132 | 0.142 |
| **Fatty Acids and Lipids** | 0.139 | 0.148 | 0.194 | 0.177 | 0.183 | 0.150 | 0.154 |
| **Potassium metabolism** | 0.108 | 0.098 | 0.125 | 0.116 | 0.126 | 0.091 | 0.103 |
| **Nitrogen Metabolism** | 0.083 | 0.086 | 0.135 | 0.113 | 0.125 | 0.073 | 0.090 |
| **Miscellaneous** | 0.070 | 0.075 | 0.089 | 0.086 | 0.080 | 0.072 | 0.074 |
| **Secondary Metabolism** | 0.013 | 0.012 | 0.015 | 0.013 | 0.015 | 0.013 | 0.012 |
| **Macromolecular Synthesis** | 0.007 | 0.006 | 0.017* | 0.010 | 0.012 | 0.008 | 0.009 |
| **Prophage** | 0.005 | 0.004 | 0.002 | 0.003 | 0.002 | 0.003 | 0.002* |
| **Photosynthesis** | 0.004 | 0.005 | 0.004 | 0.006 | 0.004 | 0.003 | 0.004 |
| **Total** | 13.656 | 13.721 | 14.222 | 14.526 | 13.412 | 11.863 | 12.740 |
