# Supplementary material for: Metagenomic and geochemical characterization of pockmarked sediments overlaying the Troll petroleum reservoir in the North Sea
Source: BMC Microbiol. 2012 Sep 11;12:203. doi: 10.1186/1471-2180-12-203 (PMC3478177; doi:10.1186/1471-2180-12-203)
Supplement: Additional file 13 — Table S8. Significantly over or underrepresented subsystems (level III) in Troll metagenomes compared to both metagenomes from the Oslofjord. Level III subsystems differing significantly in one or more Troll metagenomes compared to both Oslofjord metagenomes after statistical analysis in STAMP. [file 1471-2180-12-203-S13.docx]

### Table S8: Significantly over or underrepresented subsystems (level III) in Troll metagenomes compared to both metagenomes from the Oslofjord

Level III subsystems differing significantly in one or more Troll metagenomes compared to both Oslofjord metagenomes after statistical analysis in STAMP.

| **Underrepresented compared to the Oslofjord** | | | **Overrepresented compared to the Oslofjord** | | |
| --- | --- | --- | --- | --- | --- |
| **level I** | **level III** | **Troll samples** | **level I** | **level III** | **Troll samples** |
| **Amino Acids and Derivatives** | Ketoisovalerate oxidoreductase | Tplain and Tpm1-2 | **Amino Acids and Derivatives** | Cyanophycin metabolism | Tplain and Tpm1-2 |
| **Carbohydrates** | N-Acetyl-Galactosamine and Galactosamine Utilization | Tplain and Tpm1-2 | **Amino Acids and Derivatives** | Glutamine synthetases | Tplain and Tpm1-2 |
| **Carbohydrates** | L-ascorbate utilization (and related gene clusters) | Tplain and Tpm1-2 | **Amino Acids and Derivatives** | Methionine Degradation | Tplain and Tpm1-2 |
| **Carbohydrates** | L-fucose utilization | Tpm1-2 | **Amino Acids and Derivatives** | Choline and Betaine Uptake and Betaine Biosynthesis | Tplain and Tpm1-2 |
| **Clustering-based subsystems** | ^1^ CBSS-1T762T79.3.peg.1T2T62T | Tpm1-2 | **Carbohydrates** | Methylcitrate cycle | Tplain |
| **Clustering-based subsystems** | ^1^ CBSS-1T762T80.1T.peg.1T561T | Tplain | **Cell Wall and Capsule** | Lipid A modifications | Tplain and Tpm1-2 |
| **Clustering-based subsystems** | ^1^ CBSS-2T5731T4.1T.peg.752T | Tplain and Tpm1-2 | **Clustering-based subsystems** | ^1^ CBSS-32T0388.3.peg.3759 | Tplain |
| **Cofactors, Vitamins, Prosthetic Groups, Pigments** | Flavodoxin | Tplain and Tpm1-2 | **Clustering-based subsystems** | ^1^ CBSS-2T2T1T988.1T.peg.771T | Tplain and Tpm1-2 |
| **DNA Metabolism** | ATP-dependent Nuclease | Tpm3 | **Clustering-based subsystems** | GABA and putrescine metabolism from cluters | Tplain and Tpm1-2 |
| **DNA Metabolism** | DNA repair and recombination eukaryotic | Tplain | **Clustering-based subsystems** | ^1^ CBSS-354.1T.peg.2T91T7 | Tpm1-2 |
| **DNA Metabolism** | DNA replication, archaeal | Tplain | **Clustering-based subsystems** | ^1^ CBSS-891T87.3.peg.2T957 | Tplain and Tpm1-2 |
| **DNA Metabolism** | DNA topoisomerases, Type I, ATP-independent | Tplain | **Clustering-based subsystems** | ^1^ CBSS-31T42T69.3.peg.1T840 | Tplain and Tpm1-2 |
| **DNA Metabolism** | Late competence | Tplain | **Clustering-based subsystems** | ^1^ CBSS-3931T2T4.3.peg.2T657 | Tplain |
| **Membrane Transport** | ECF class transporters | Tplain and Tpm1-2 | **Clustering-based subsystems** | ^1^ CBSS-2T0592T2T.3.peg.1T809 | Tplain, Tpm1-1 and Tpm1-2 |
| **Motility and Chemotaxis** | Archaeal Flagellum | Tplain, Tpm1-1, Tpm1-2, Tpm2 and Tpm3 | **Cofactors, Vitamins, Prosthetic Groups, Pigments** | Chlorophyll Degradation | Tpm1-2 |
| **Nitrogen Metabolism** | Nitrogen fixation | Tpm2 | **Fatty Acids and Lipids** | carnitine metabolism | Tpm1-2 |
| **Nitrogen Metabolism** | Nitrosative stress | Tpm1-2 | **Macromolecular Synthesis** | ^1^ CBSS-2T432T77.1T.peg.51T1T | Tplain |
| **Protein Metabolism** | Glu-tRNA(Gln) transamidation | Tplain | **Metabolism of Aromatic Compounds** | Biphenyl Degradation | Tplain and Tpm1-2 |
| **Protein Metabolism** | Ribosome LSU eukaryotic and archaeal | Tplain | **Metabolism of Aromatic Compounds** | Phenylpropanoid compound degradation | Tplain and Tpm1-2 |
| **Protein Metabolism** | Ribosome SSU eukaryotic and archaeal | Tplain | **Metabolism of Aromatic Compounds** | Benzoate transport and degradation cluster | Tplain |
| **Protein Metabolism** | Translation elongation factors eukaryotic and archaeal | Tplain | **Metabolism of Aromatic Compounds** | Gentisare degradation | Tplain and Tpm1-2 |
| **Regulation and Cell signaling** | Coagulation cascade | Tpm1-2 | **Metabolism of Aromatic Compounds** | Salicylate and gentisate catabolism | Tplain and Tpm1-2 |
| **Respiration** | V-Type ATP synthase | Tplain | **Miscellaneous** | Biolumenescence | Tplain and Tpm1-2 |
| **Respiration** | Carbon monoxide induced hydrogenase | Tplain and Tpm1-2 | **Nitrogen Metabolism** | Ammonia assimilation | Tplain |
| **Respiration** | Reductive Dechlorination | Tpm1-2 | **Nitrogen Metabolism** | Dissimilatory nitrite reductase | Tpm1-2 |
| **RNA Metabolism** | Wyeosine-MimG Biosynthesis | Tpm1-2 | **Nitrogen Metabolism** | Nitric oxide synthase | Tplain, Tpm1-2 and Tpm2 |
| **RNA Metabolism** | RNA polymerase archaeal | Tplain | **Regulation and Cell signaling** | DNA-binding regulatory proteins, strays | Tplain and Tpm1-1 |
| **RNA Metabolism** | RNA polymerase archaeal initiation factors | Tplain | **Regulation and Cell signaling** | Orphan regulatory proteins | Tplain and Tpm1-2 |
| **RNA Metabolism** | RNA polymerase II | Tplain, Tpm1-2 and Tpm3 | **Respiration** | Terminal cytochrome C oxidases | Tplain and Tpm1-2 |
| **Stress Response** | Rubrerythrin | Tpm1-2 | **Respiration** | Biogenesis of cytochrome c oxidases | Tplain and Tpm1-2 |
| **Unclassified** | Acetone Butanol Ethanol Synthesis | Tplain and Tpm1-2 | **Stress Response** | Protection from Reactive Oxygen Species | Tplain and Tpm1-2 |
| **Unclassified** | CR clusters Euk 1T | Tplain and Tpm1-2 | **Stress Response** | Flavohaemoglobin | Tplain |
| **Virulence** | Adhesion of Campylobacter | Tpm1-2 | **Sulfur Metabolism** | Inorganic Sulfur Assimilation | Tplain |
| **Virulence** | Heme, hemin uptake and utilization systems in GramPositives | Tpm2 | **Unclassified** | Acyclic terpenes utilization | Tplain and Tpm1-2 |
| **Virulence** | Pseudaminic Acid Biosynthesis | Tplain, Tpm1-2 and Tpm3 | **Unclassified** | PQQ-dependent quinoprotein dehydrogenases | Tplain and Tpm1-2 |
| **Virulence** | Bacterial Endolysins: autolysins, phage, and phage-like lysins | Tpm1-2 | **Unclassified** | YgfZ-Fe-S clustering | Tplain and Tpm1-2 |
| **Virulence** | Listeria phi-A1T1T8-like prophages | Tpm1-2 | **Virulence** | Hemin transport system | Tplain and Tpm1-2 |
| **Virulence** | Staphylococcal phi-Mu50B-like prophages | Tpm1-2 |  |  |  |
| **Virulence** | Arsenic resistance | Tplain |  |  |  |
| **Virulence** | Streptolysin S Biosynthesis and Transport | Tpm1-2 |  |  |  |

^1^ Clustering-based subsystems: subsystems in which there is functional coupling evidence that genes belong together, but their exact function is not known.
