# Supplementary material for: Metagenomic and geochemical characterization of pockmarked sediments overlaying the Troll petroleum reservoir in the North Sea
Source: BMC Microbiol. 2012 Sep 11;12:203. doi: 10.1186/1471-2180-12-203 (PMC3478177; doi:10.1186/1471-2180-12-203)
Supplement: Additional file 14 — Methods for geochemical data. Methods used to obtain geochemical data [25]. [file 1471-2180-12-203-S14.docx]

### Methods for geochemical data

Methods used to obtain geochemical data [[25](#_ENREF_24)].

Hydrocarbon/TOC

All procedures were performed according to NIGOGA, 4^th^ edition, carried out by Applied Petroleum Technology (Kjeller, Norway).

Pore water

Sediment pore water was sampled by centrifugation. Electric conductivity was measured immediately according to NS-ISO 7888 by the Norwegian Geotechnical Institute (Oslo, Norway).

Analytica (Oslo, Norway) performed the metal analyses according to (modified) EPA methods 200.7 and 200.8.

Bioforsk (Ås, Norway) performed the following analyses:

Hydrogen carbonate

100 ml of the sample was titrated with 0.1 M HCl until pH 4.5 was reached and the quantity of bicarbonate calculated.

Ammonium-N

Berthelot’s reagent was used and absorbance was measured at 630 nm using an autoanalyzer.

Nitrite-N + Nitrate-N

The nitrite content was measured in an auto analyzer. A reaction with sulfanilamide and N-1-naphtyl ethylene diamine for creation of a colored complex with absorbance at 550 nm was used.

Nitrate was reduced to nitrate in a copper/cadmium reducer at pH 8. The resulting nitrate was then analyzed as previously described.

Chloride and Sulphate-S

Measurement of chloride and sulphate-S was based on NS-EN ISO 10304-1, using an ion chromatograph.
